# VP2-TALE fusions increase AAV2 capsid filling with payload bearing cognate TALE target sequences as determined by qPCR/ELISA

**DOI:** 10.1016/j.omta.2026.201877

**Published:** 2026-10-26

**Authors:** Qiantong Chen, Mehtap Bal, Geoffrey Howe, Robert Whitfield, Darren N. Nesbeth

**Affiliations:** 1 Department of Biochemical Engineering, University College London, Bernard Katz Building, London WC1E 7JE, UK; 2 Cytiva Life Sciences, Amersham Place, Little Chalfont, Amersham HP7 9NA, UK; 3 Institute of Neuropathology, University Hospital Zurich, Schmelzbergstrasse 12, 8091 Zurich, Switzerland

**Keywords:** synthetic biology, gene therapy, AAV2, cell engineering

## Abstract

We sought to improve the empty/full ratio of recombinant adeno-associated virus serotype 2 (AAV2) production by engineering the capsid to increase the favorability of its interactions with payload genomes. We fused a transcription activator-like effector (TALE), sequence-specific DNA-binding domain to the VP2 protein (VP2-TALE), either directly or via flexible linkers, within a transgene encoded by an overexpression plasmid. We also engineered seven different versions of the green fluorescent protein (GFP) payload plasmid, with up to three copies of a 14 base-pair TALE-cognate target sequence. Co-transfection with a helper plasmid, a RepCap plasmid engineered to prevent native VP2 expression, and a VP2-TALE expression plasmid resulted in lower capsid titers and higher viral genome titers when TALE target sequences were present in the payload plasmid than in control transfections in which the payload lacked TALE target sequences. Western blotting with an anti-VP antibody showed VP-positive proteins of the size predicted for VP2-TALE fusion. Electron microscopy indicated the presence of correctly formed viral capsids. ELISA and qPCR analysis indicated that these transfections could result in increases in capsid filling approaching 200%.

## Graphical abstract

**Figure:**
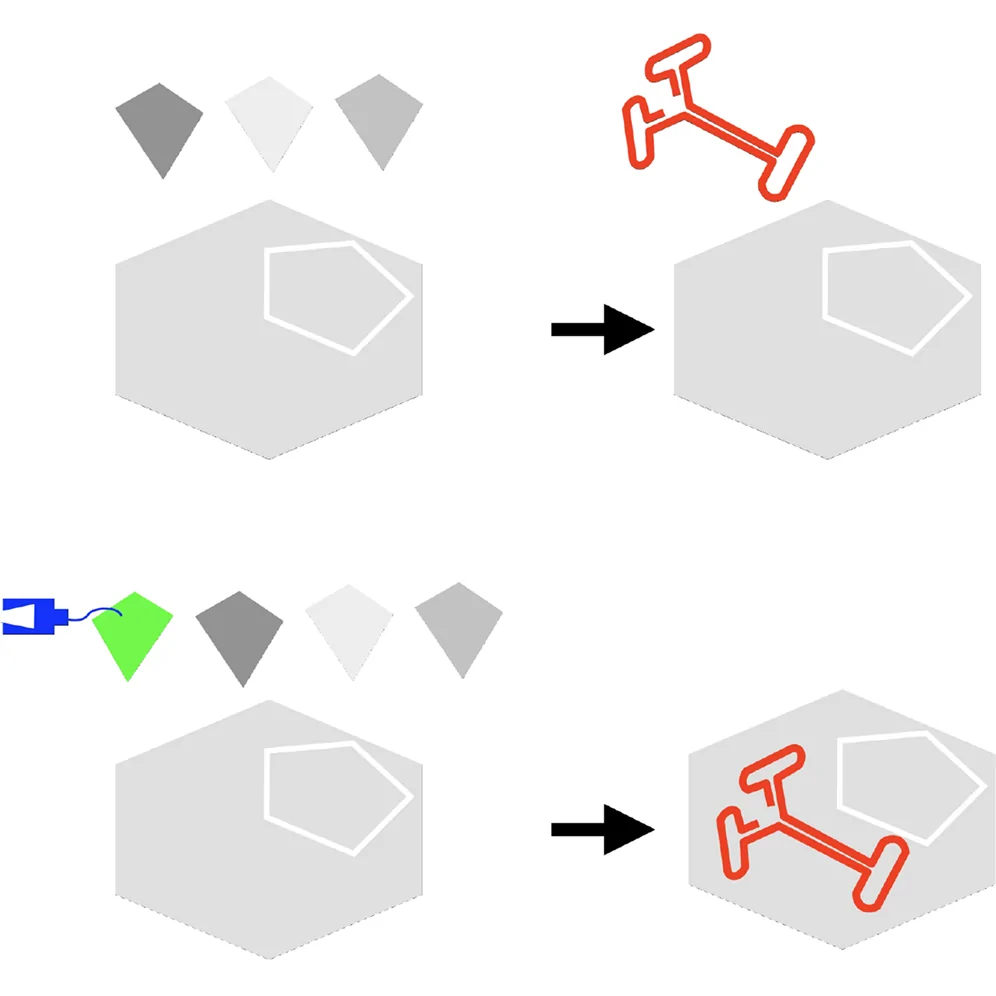

## Teaser

Empty capsids dog AAV2 biomanufacturing. Nesbeth and colleagues genetically fused the AAV2 capsid protein, VP2, to a transcription activator-like effector (TALE) domain while in parallel inserting cognate TALE-target DNA sequences into the payload genome. This resulted in 200% increases in capsid filling, as indirectly estimated using qPCR and ELISA.

## Introduction

Recombinant adeno-associated virus (AAV) vectors have emerged as leading vehicles for gene therapy applications due to their generally low immunogenicity and high levels of tolerability in patients.^1^^,^^2^ AAV can also be used to transduce both dividing and non-dividing cells with long-term expression of delivered transgenes.^3^^,^^4^ AAV serotype 2 (AAV2) is effective for gene delivery to neurons and retinal cells, lending it potential as a gene therapy tool for addressing genetic diseases of the central nervous system and eyes.^5^^,^^6^ However, AAV vector manufacturing faces significant challenges, particularly in achieving high genome-packaging efficiency during production. Current AAV production platforms typically achieve suboptimal packaging, with a substantial proportion of empty capsids that reduce therapeutic potency and increase immunogenicity concerns.^7^^,^^8^^,^^9^

AAV particles are icosahedra formed by the AAV “VP” capsid proteins, VP1, VP2, and VP3. Becerra et al. reported a ratio of 1:10 between each of VP1 and VP2 and the more abundant VP3 protein (Figure S1A), indicating a 5:5:50 ratio for particles composed of 60 VP subunits.^10^ However, native mass spectrometry data reported by Wörner et al.^11^ indicated that AAV icosahedra may actually assemble stochastically, leading to a highly heterogeneous population of capsids of variable VP composition, such that virtually every capsid in a given preparation may have a unique stoichiometry.

As icosahedra, AAV particles have 2-fold, 3-fold, and 5-fold symmetry axes (Figure S1B). AAV proteins Rep78 and Rep68 bind to inverted terminal repeat (ITR) sequences present within viral payload genomes and anchor near the centers of the 5-fold symmetry axes.^12^ AAV proteins Rep52 and Rep40 then direct viral genome insertion into capsids in an ATP-dependent manner.^13^^,^^14^^,^^15^ Rep78 and Rep68 (Rep78/Rep68) are involved in multiple steps of the AAV life cycle, including AAV genomic DNA replication and integration into host-cell genome, as well as capsid packaging. Sequence-specific binding of Rep78/Rep68 to payload DNA determines association with the stem,^16^ loop of the stem-loop (hairpin) structure of each AAV ITR,^17^ and also the position within the AAV genome where they mediate cleavage via an endonuclease activity.^18^ Sequence-specific AAV genome binding by Rep68/Rep78 involves an extensive range of oligomerization states.^19^ Despite this, mechanistic studies also indicate that this bindings exhibits a high degree of structural flexibility, likely due to the multiple different functions served by the binding.^20^

The *rep* gene has been extensively engineered to improve AAV bioprocessing. Changes to Rep protein post-translational modification (PTM) sites improved AAV2 capsid filling.^21^ Jain et al. identified single codon variants of the Rep gene that conferred 50%–100% improvements in nuclease-protected AAV particle titer.^22^ Inter-serotype, domain-swapped Rep protein variants also improved capsid filling for multiple serotypes,^23^ and screening of a multi-serotype, directed evolution Rep library identified variants able to effect up to 2.5-fold improvement in capsid filling.^24^ The DNA sequence of the p19 promoter region, which not only controls Rep52/40 transcription but also sits within the wider Rep open reading frame (ORF), can also be varied in a manner that influences genome filling but does not alter the encoded amino acid residue sequence.^25^ However, understanding of the precise molecular events that define AAV capsid assembly and genome packaging remains incomplete.^26^^,^^27^^,^^28^ It is broadly understood that these processes occur on the basis of VP proteins, Rep proteins, and viral genomes undergoing Fickian diffusion within the crowded intracellular environment (Figure S1C).^29^ Collision and association of these objects is therefore a stochastic process^30^^,^^31^^,^^32^ with the stability of the final icosahedron structure being a factor that favors its formation.^33^

In summary, Rep proteins, payload DNA, and capsids, as separate objects, must stochastically diffuse and collide (Figure S1C). Upon association, evolved regions of affinity between capsid and Rep proteins, and between Rep proteins and payload DNA, then favor subsequent procedures that result in payload upload into the capsid interior. Here, we sought to bias these stochastic processes in favor of payload genome upload by engineering an affinity between capsid and payload. We anticipated that this engineered affinity would result in Rep proteins encountering both free payload DNA and capsid-associated payload DNA.Crucially, our assumption was that capsid-associated payload DNA would still be compatible with Rep-mediated genome-to-capsid upload procedures, given the structural flexibility of Rep-to-genome interactions.^20^ As such, our approach may also be used alongside the strategies we discussed earlier to improve AAV capsid filling via Rep gene modification in future.

As well as being the joint-least abundant component of the AAV capsid (along with VP1), VP2 protein has been shown to be tolerant of N-terminal fusions with exogenous protein domains.^34^ Such fused domains tend to be displayed on the exterior of capsid particles, which also retain the ability to properly assemble.^35^ Here, we sought to improve the level of AAV2 capsid filling with a novel set of changes to both the VP2 protein and payload genome (Figure 1), predicated on increasing the level of association between capsids and payload genomes. For this study, we sought to characterize novel variants solely in terms of the important bioprocess metrics of (1) viral genome productivity, (2) infectiousness, and (3) indirect inference of capsid filling. We anticipated that those variants identified to have the best bioprocess performance would be the most useful candidates for future studies, beyond the scope of this current study, where structural and binding properties of components could be directly characterized.

**Figure 1:**
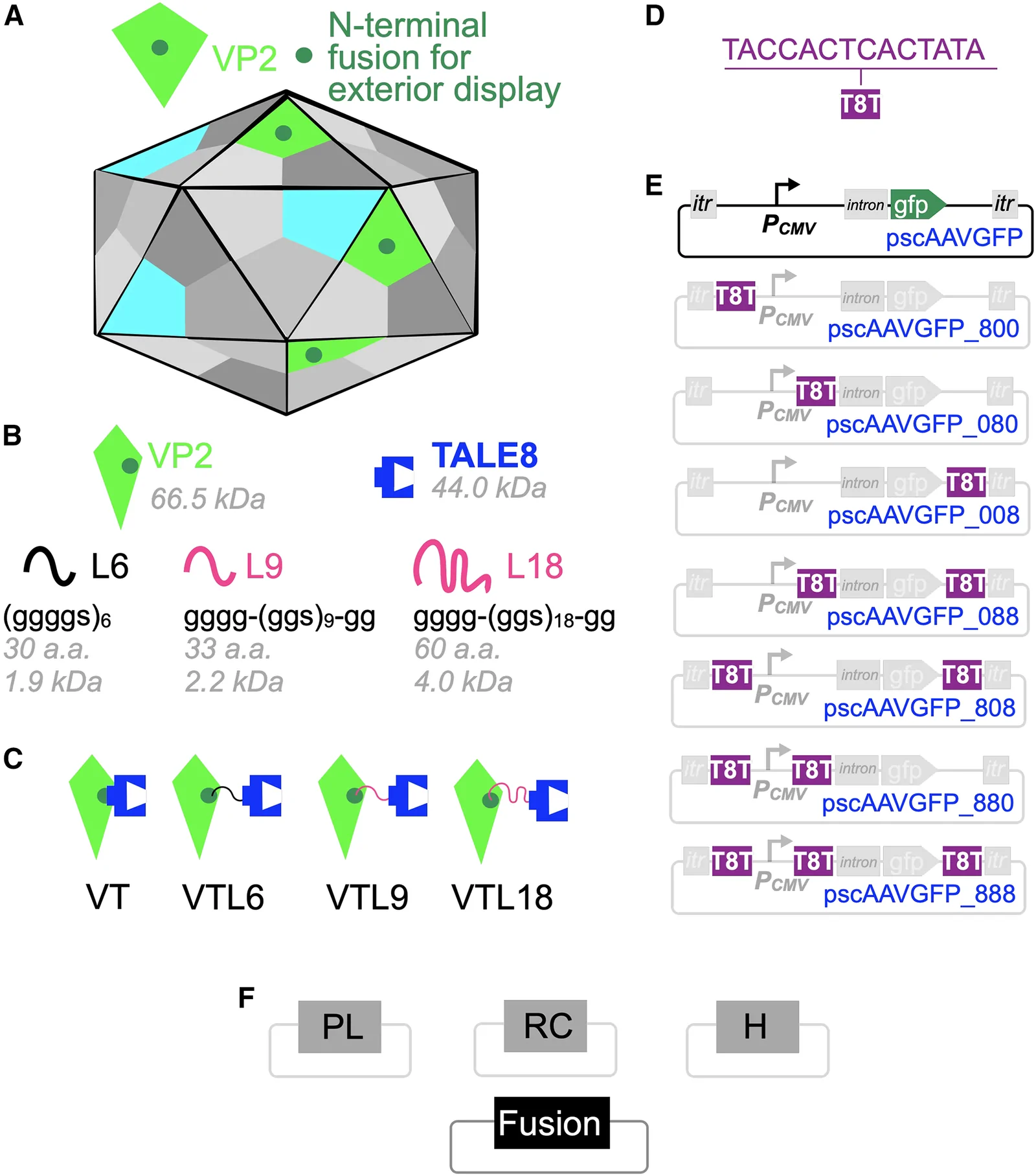
Design sketch of VP2-TALE fusion proteins and AAV2 payload constructs (A) Schematic representation of VP2 capsid protein with N-terminal fusion site designated for exterior display of fusion partners. (B) Components used for fusion protein designs: VP2 (green, 66.5 kDa), TALE8 DNA-binding domain (blue, 44.0 kDa), and linkers of varying lengths— L6 (30 amino acids, 1.9 kDa), L9 (33 amino acids, 2.2 kDa), and L18 (60 amino acids, 4.0 kDa). (C) Four fusion protein designs: VT (direct fusion), VTL6, VTL9, and VTL18 (fusions with different linker lengths). (D and E) (D) TALE8 target (T8T) sequence (E) AAV payload plasmid constructs containing GFP reporter gene, featuring the strong, constitutive CMV promoter, an intron, and the GFP ORF, with various configurations of T8T sequence inserts (purple boxes) intended for TALE8 binding at different positions (800, 080, 008, 088, 808, 880, and 888). (F) Illustration of the four-plasmid transfections used in this study, which feature the standard AAV plasmid types: PL, payload; RC, RepCap; H, helper, plus, for stated transfections, Fusion = one of four different plasmids encoding expression of VP2-TALE fusion proteins.

We designed four fusion proteins (Figures 1A–1C) combining the VP2 capsid protein with a transcription activator-like effector (TALE) domain specifically designed to recognize and bind predetermined sequences engineered into the payload genome (Figures 1D and 1E). TALEs are modular bacterial proteins that can be customized to bind DNA on a sequence-specific basis.^36^^,^^37^^,^^38^ Here, we used the sequence of a TALE DNA-binding domain, referred to here as “TALE8”, previously used by Zhang et al.^39^ in the context of a synthetic transcription factor, where the 14 base pair (bp) target sequence, TACCACTCACTATA, was present in a synthetic promoter site.

We anticipated the possibility that a TALE domain directly fused to VP2 may not have sufficient steric freedom to bind payload genomes in a manner that still favors their transfer to the capsid interior. As such, we designed transgenes encoding VP2-TALE fusion proteins with no linker and linkers differing in length and composition, to ascertain if linker presence or length influenced capsid filling. All three flexible linker sequences used here (Figure 1B) had previously been shown to be effective for favoring native association of the components of multi-protein immune complexes.^40^

We sought to balance the potential benefit of adding TALE-binding sites to the payload genome with the need to minimize the number of changes made to the payload sequence, preserving its capacity to encode therapeutic payloads. As such, we incorporated a maximum of three copies of the TACCACTCACTATA (Figure 1D) cognate TALE target site, referred to here as “T8T”, within the viral payload genome (Figure 1E).

Our hypothesis was that combining payloads with T8T targets with plasmids encoding VP2-TALE fusion proteins (Figure 1C) could together improve capsid filling while preserving infectiousness. We envisaged a scenario in which the TALE-to-target binding would affect a relative increase in the net number of capsid:genome encounters per unit time, and consequently an increase in the percentage of capsids overall that are filled with payload genomes. We also wished to determine the extent to which the presence of the 44 kDa TALE8 domain (Figure 1C) on the viral exterior was compatible with acceptable levels of payload filling and/or viral infectiousness. Our approach builds upon previous successful capsid engineering efforts for improved clinical efficacy, including the incorporation of targeting ligands and cell-penetrating peptides into AAV capsids,^41^^,^^42^ while addressing the distinct bioprocess challenge of capsid filling efficiency.

## Results

Plasmids pTALEVP_01, pTALEVP_L6, pTALEVP_L9, and pTALEVP_L18 (Figure S2) were designed and assembled to encode the following VP2-TALE8 fusion proteins: VT (linkerless), VTL6, VTL9, and VTL18, respectively (Figures 1B and 1C), with expression driven by the CMV promoter. The plasmid pscAAV-GFP (Addgene plasmid #32396) encodes a self-complementary AAV payload genome consisting of a green fluorescent protein (GFP) expression cassette.^43^ Seven variants of pscAAV-GFP were assembled, each incorporating one, two or three copies of the TACCACTCACTATA, 14-bp TALE8 cognate DNA target sequences (T8T) across three defined insertion sites within the payload genome (Figures 1D and 1E). These insertion sites were chosen to fall outside any other genetic element, such as the promoter, ORF, intron, or the ITRs, which are understood to be bound by Rep proteins during capsid filling.^44^ These seven payload variants were abbreviated, according to the presence and position of the T8T targets, as “888” (T8T sequences at all three sites) or as “008”, “080”, “800”, “808”, “088”, or “880” for the other configurations (Figure 1E).

### Impact on AAV2 production of inserting T8T target sequences into payloads

We first sought to isolate the impact of the presence of the T8T insert sequences within payload plasmids on the performance of a standard AAV2 transient transfection production procedure. To this end, we performed standard transfections (helper, RepCap, and payload plasmids) while swapping the original, pscAAV-GFP, payload for one of the seven T8T payload variants (Figure 1E). For all seven T8T insert payloads variants, and the unmodified payload, the resulting yield performance did not differ to a statistically significant extent when determined over *n* = 3 biological repeats for viral genomes per mL (vg/mL) (Figures 2A and 2B), *n* = 2 biological repeats for transducing units per mL (TU/mL) (Figures 2C and 2D), and the mean fluorescence intensity (MFI) of the transduced cells (Figure S4). The quantitative PCR (qPCR) procedure for vg/mL determination included a nuclease treatment to degrade unencapsulated viral genomes. The lack of statistically significant differences between vg/mL yields (Figures 2A and 2B) is consistent with, but not definitive proof of, the fact that the presence of T8T sites in payload genomes did not compromise their ability to be associated with capsids in a nuclease-resistant manner.

**Figure 2:**
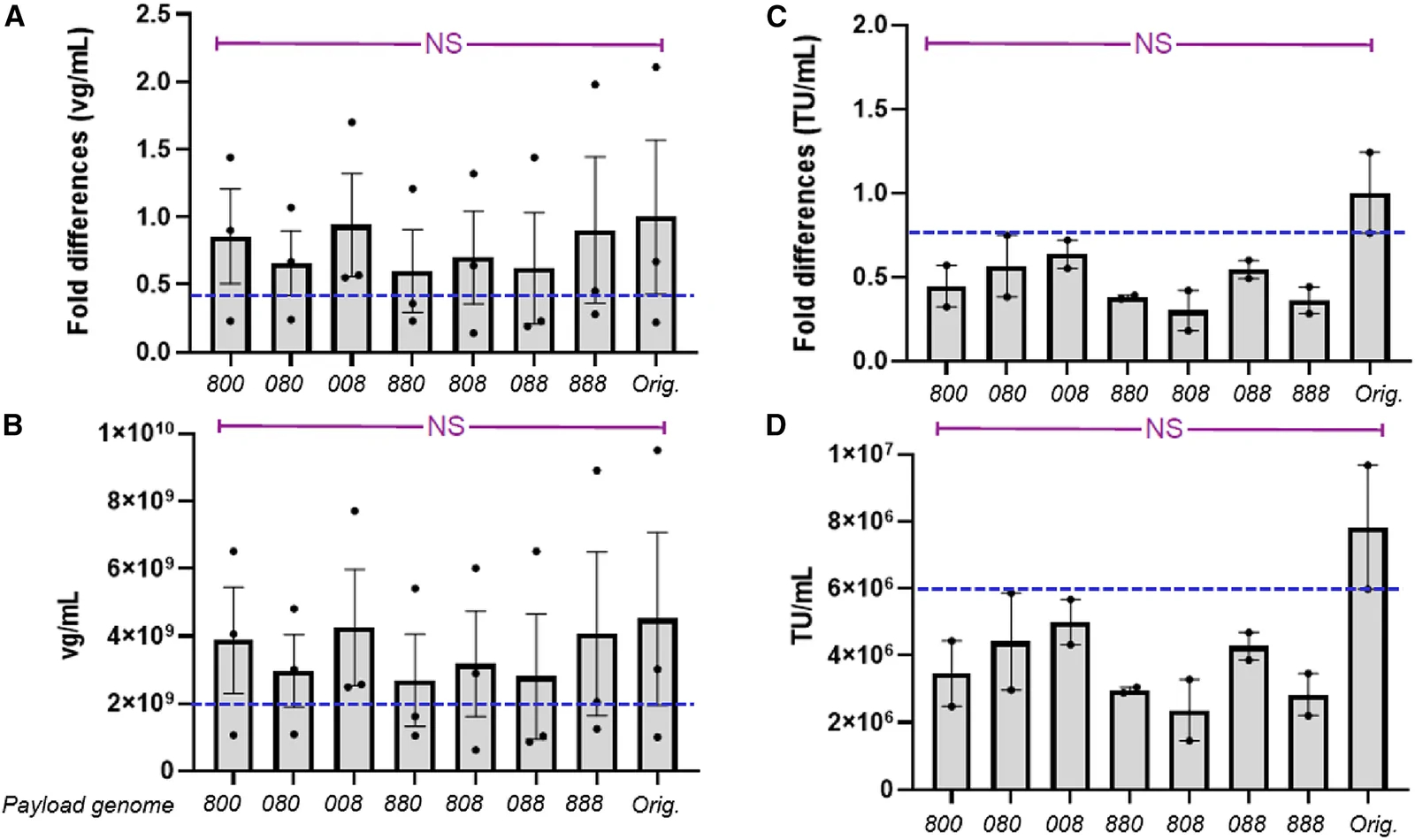
AAV2 vg/mL and TU/mL yields as a function of number and location of TALE8 target sequence(s) within payload plasmid Transfection for AAV2 production was performed at 1 mL scale as described in materials and methods. Each transfection featured either the original, unmodified payload plasmid, pscAAVGFP (data bars labeled “Orig.”), or one of the seven engineered payload plasmids detailed in Figure 1E, abbreviated here to 800, 080, 008, 880, 808, 088, or 888 for data bar labels. For all yields the Brown-Forsythe ANOVA and Dunnett’s T3 post hoc tests were applied to determine if any yield differed from any other yield to a statistically significant degree. All resulting *p* values were >0.05 and therefore deemed to be non-significant (NS), and so all data was placed under a bracket labeled ”NS”. (A) Fold differences in viral genome yields (vg/mL) between transfections featuring the engineered payload plasmids versus the unmodified payload plasmid. (B) Resulting vg/mL performance of each transfection. All data in plots (A) and (B) are mean values with error bars of plus and minus the standard error of the mean (±SEM) from three biological replicates, with determinations made via 2–4 qPCR measurements per biological repeat. (C) Fold differences in transducing unit yields (TU/mL) between transfections with engineered payload versus the unmodified payload. (D) Resulting TU/mL performance for each transfection. All data in plots (C) and (D) are mean ± SEM from two biological replicates, with determinations made via 2–3 viral transductions and flow cytometry measurements per biological repeat. Blue dotted line highlights lower SEM limit for the yield from standard transfection.

The TU/mL yields (Figures 2C and 2D) and the MFI (Figures S4A and S4B) revealed no correlative effect of the position of the 14 bp insertion, comparing yields using 008, 080, and 800 payload plasmids, and no correlative effect from the number of insertions—comparing yields from the single-insert payload plasmids to the double-insert plasmids (088, 808, and 880) and triple insert plasmid (888). The payload plasmid with the highest number of T8T inserts (888, encoded by pscAAVGFP_888) yielded vg/mL and TU/mL levels that did not differ from those arising from standard production to a statistically significant degree. As such, we elected to use pscAAVGFP_888 for the rest of the investigation.

### Swapping RepCap plasmid for plasmids encoding only VP2-TALE8 fusion proteins

We next performed standard, three-plasmid transfections but entirely omitting the original, pAAV-RC2, RepCap plasmid and replacing it with one of the four plasmids encoding VP2-TALE8 fusion proteins (Figure 1C) to determine whether the fusion alone could be sufficient for AAV2 production. The TU/mL yield achieved by the standard procedure (Figure 3Ai, data bar 1) was completely abolished in all transfection procedures lacking the RepCap plasmid (Figure 3Ai, data bars 2–5). Substitution of the pscAAV-GFP payload plasmid for pscAAVGFP_888 similarly abolished TU/mL yield (Figure 3Ai, data bars 6–9). None of these attempts at transduction (Figure 3Ai, data bars 2–9) resulted in a percentage of fluorescent target cells that was greater than the background autofluorescence observed for targets cells incubated with lysate material arising from untransfected cells (data bar 10, Figure 3Ai–ii), which we treated as a limited of blank (LOB) for this assay.

**Figure 3:**
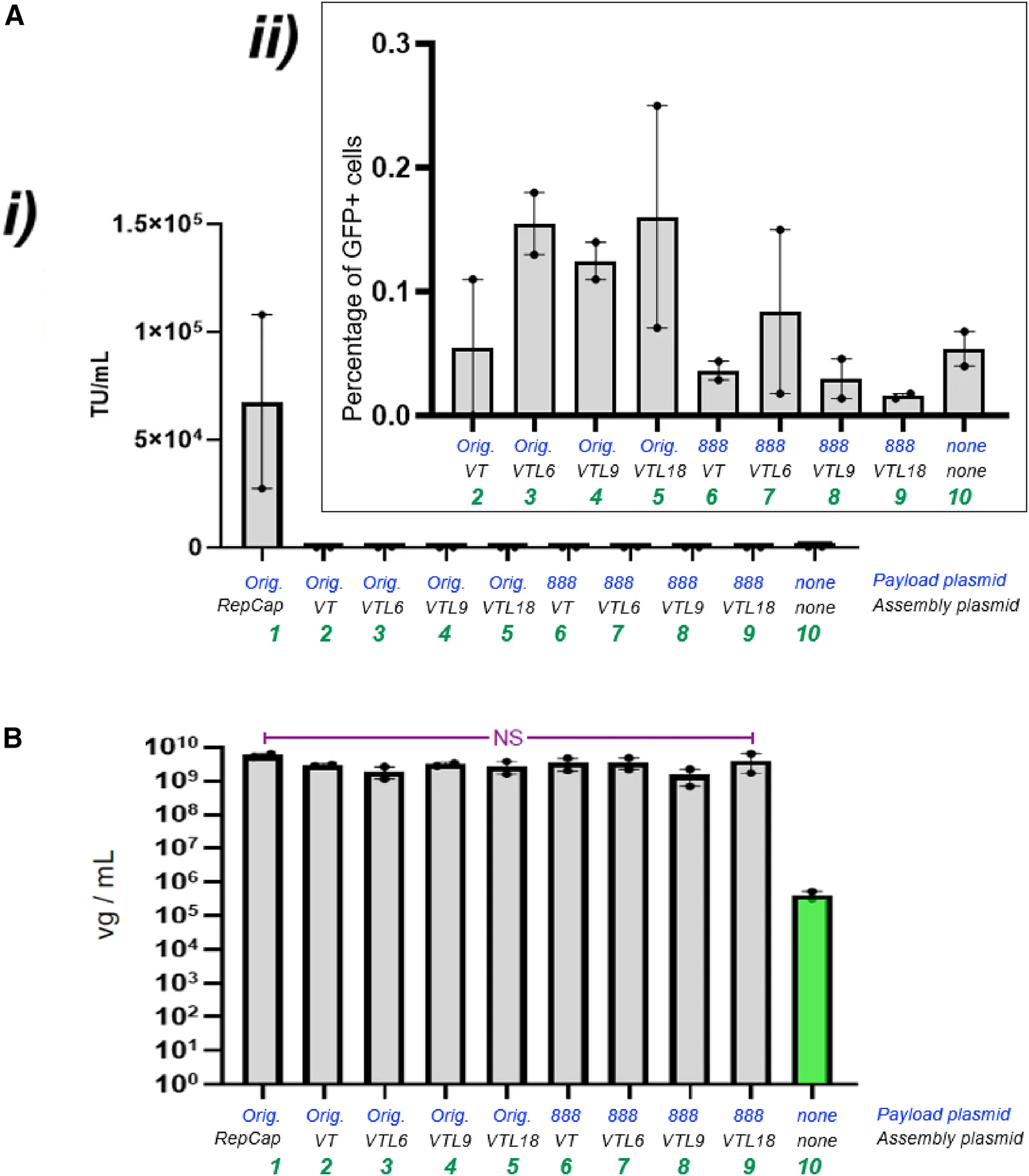
Impact of replacing the RepCap plasmid with a plasmid encoding only a VP2-TALE fusion on AAV2 infectiousness and vg/mL yield AAV2 production was performed via transfection at 1 mL scale using standard Helper plasmid plus (blue text) either the original payload plasmid, pscAAVGFP (“Orig.”), or the engineered payload, pscAAVGFP_888 (Figure 1E, lowermost diagram). For capsid assembly (black text), either the standard RepCap plasmid, pAAV-RC2, or one of four plasmids encoding VP2-TALE8 fusion proteins (Figure 1C) were used. (A) (i) Transducing units per mL (TU/mL) were determined by flow cytometry. Error bars represent ±SEM from two AAV2 production transfections (biological repeats), with determinations made via two transduction procedures and flow cytometry measurements per biological replicate. The “none” control (rightmost data bar) data is from HEK293T target cells incubated with cell lysate from untransfected HEK293-F cells. While the standard transfection (leftmost data bar) yielded measurable titer, all experimental transfections resulted in a percentage of GFP-positive cells below the 1% threshold needed for meaningful or accurate titer determination. Due to the clarity of the result, no statistical tests were applied. (A) (ii) For transduction procedures 2–10 in (A) (i) data, cells in which fluorescence was detected was plotted as a percentage of total cells. (B) Viral genome titer (vg/mL) was determined via qPCR for lysates arising from cells that had undergone transfection for AAV production (data bars 1–9) and untransfected cells (green data bar 10 labeled “none”) as a negative control and limit of quantitation. Data are mean ± SEM from two biological repeats, with determinations made via four qPCR measurements per biological repeat. The vg/mL titers arising from transfected cells (bars 1–9) were all at least three orders of magnitude higher than the none control, but did not differ between each other to a statistically significant degree, as tested by Brown-Forsythe ANOVA and Dunnett’s T3 post hoc tests (indicated by bracket labeled NS for non-significant).

By contrast, when the RepCap plasmid was either retained or replaced with VP2-TALE8 fusion plasmids, the resulting vg/mL yields (Figure 3B, data bars 1–5) did not differ from each other to a statistically significant extent. Additional substitution of pscAAV-GFP payload plasmid for pscAAVGFP_888 had a similar lack of effect on vg/mL yield (Figure 3B, data bars 6–9). This preservation of vg/mL yield performance indicated that the VP2-TALE8 fusion proteins alone could associate with payload genomes in a manner sufficient to confer resistance to the nuclease incubation step of the qPCR procedure (materials and methods). However, the observed effect on TU/mL suggested that, when present as the sole capsid protein, the VP2-TALE8 fusions could not confer the ability to transduce target cells, regardless of the presence or absence of T8T target sequences in payload genomes.

### Including VP2-TALE8 fusion expression plasmids in four-plasmid transfections

We next tested whether providing both a VP2-TALE8 fusion expression plasmid and a RepCap plasmid, in a four-plasmid procedure (Table S1; Figure 4), could support or improve transduction-competent AAV2 capsid production. We performed a set of four-plasmid transfections using the same 1.5 μg total plasmid mass as used previously for three plasmid transfections (Figure 3). A 1:1:1:1 molar ratio was used for Helper:Payload:RepCap:Fusion plasmids and also for variants of payload and RepCap. Where indicated in Figure 4, for some transfections the molar ratio of the RepCap (and variant) and VP2-TALE8 fusion protein expression plasmid (Figure S2) was varied to 1.5:0.5 or 1.8:0.2 (and the reverse) while the molar ratio of the other two plasmids, and overall plasmid DNA mass, was unchanged. As such, these four-plasmid transfections featured a lower absolute mass of helper and payload than in the classic three-plasmid transfection, and, depending on the molar ratios used, a higher or lower absolute mass of RepCap plasmid (Table S1). We envisaged a scenario in which the nuclease protection observed for VP2-TALE8 fusion protein expression plasmids, evidenced in Figure 3B, could be combined with the infectiousness level that was observed when RepCap and the T8T-insert payload plasmids were used in transfections (Figures 2C and 2D). We speculated that resultant yield improvements might be due to AAV2 capsids, formed by native VP1, VP2, and VP3 proteins, incorporating one or more copies of the VP2-TALE8 fusion.

**Figure 4:**
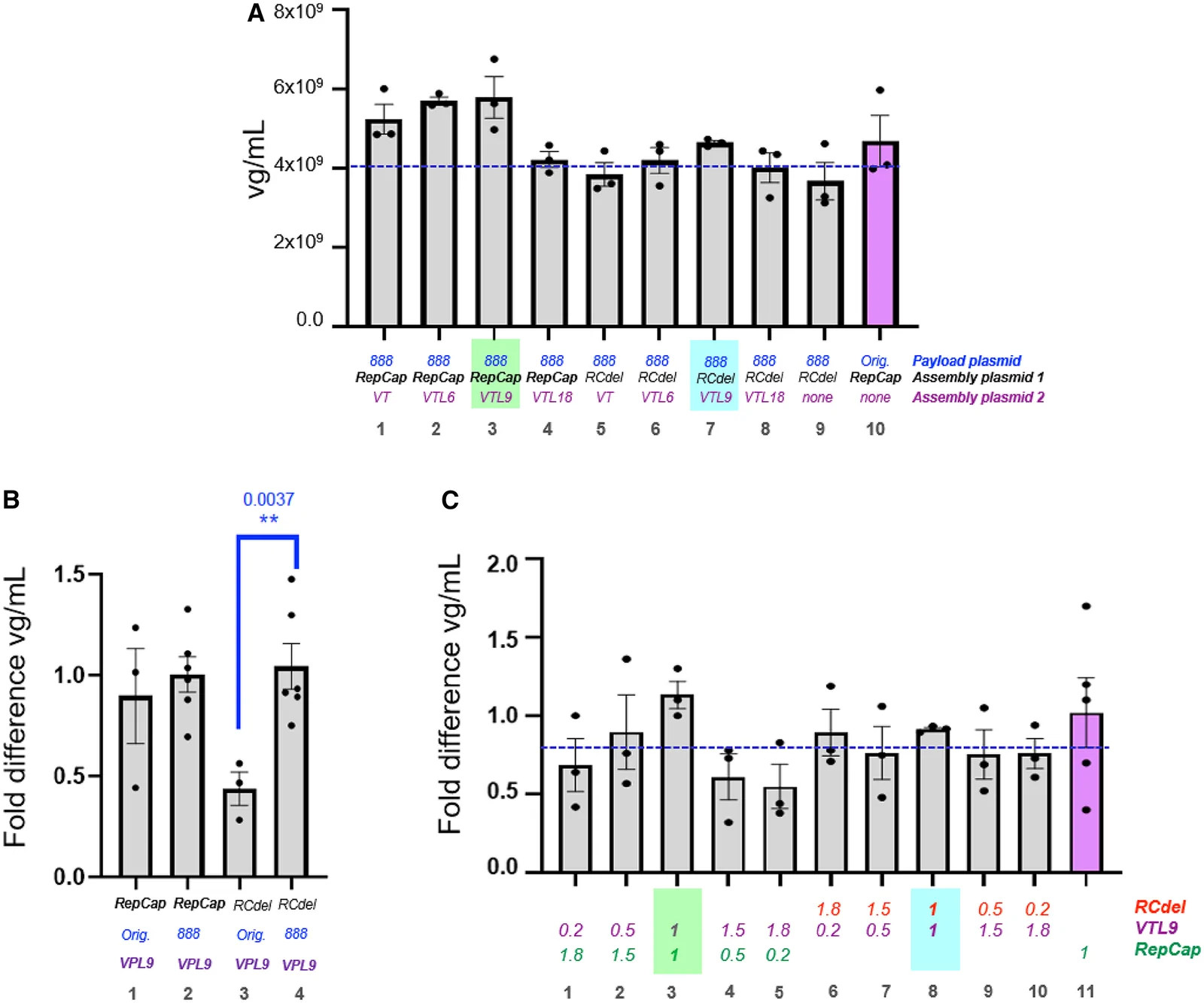
Impact on vg/mL yield of including VP2-TALE8 expression plasmids and T8T payload plasmids within four-plasmid AAV2 production AAV2 production was performed at 1 mL scale using either four-plasmid transfections or three-plasmid transfections as controls. Briefly, all transfections included helper plasmid plus a plasmid encoding original payload (Orig.) or payload 888 (Figure 1D), plus either original RepCap plasmid or a version of the RepCap modified to prevent native VP2 production (RCdel). Indicated transfections included, as a fourth plasmid, one of four plasmids encoding VP2-TALE8 fusion proteins (Figure 1C). For (A) and (C), data are mean ± SEM from at least three biological replicates, with vg/mL determinations made via two qPCR measurements per biological repeat. Also, for (A) and (C), a Brown-Forsythe ANOVA and Dunnett’s T3 post hoc tests was applied to compare each experimental transfection type (gray data bars) to the standard transfection data (purple data bar). All data bars are annotated with a *p* value or bar bracket only if a statistically significant difference of *p* < 0.05 (⁎) or *p* = between 0.01 and 0.001 (⁎⁎) arose and left unannotated if there was no statistically significant difference to comparator. (A) Resulting vg/mL yields from transfections using indicated plasmid combinations. Dotted horizontal line highlights lower SEM limit for the purple data bar. (B) Fold difference between vg/mL resulting from transfections using indicated plasmids and the vg/mL resulting from the standard, three-plasmid transfection (data bars, 10 in plot A). All data are mean ± SEM from three to six biological replicates, with determinations made via two qPCR measurements per biological repeat. A two-tailed Welch’s *t* test indicated no statistically significant difference between the leftmost pairs of means, hence no annotation, but an indicated (blue text) level of difference between the rightmost pair. (C) Fold difference as in plot (B) but using indicated molar ratios of the plasmid encoding the VTL9 fusion protein and either RepCap or RCdel. Values from the plot (A) data bars 3, 7 (highlighted in green and cyan respectively) are re-plotted in plot (C) (data bars 3 and 8, respectively). Dotted horizontal line highlights lower SEM limit for the purple data bar.

### Impact of VP2-TALE8 fusion on vg/mL yield from four-plasmid transfections

We first used the standard RepCap, helper, and pscAAVGFP_888 plasmids along with each of the four plasmids (Figure S2; Table S1) encoding VP2-TALE8 fusion proteins (Figure 4A, gray data bars 1–4). The resulting vg/mL yields did not differ from that of standard, three-plasmid AAV2 production (Figure 4A, purple data bar) to a statistically significant degree. We repeated these transfections but substituted the pAAV-RC2 RepCap plasmid with its modified version, pRep-VP1/3 (abbreviated to “RCdel”), featuring the ACC-to-ACG Cap gene mutation shown previously^45^^,^^46^ to prevent production of VP2, while preserving ability to direct expression of VP1 and VP3 ORFs (Figure S2). The resulting vg/mL yields (Figure 4A, gray data bars 5–8) again did not significantly differ from the yield resulting from standard, three-plasmid AAV2 production (Figure 4A, purple data bar). These yields also did not significantly differ from the yield resulting from a three-plasmid AAV2 production in which RepCap was swapped for RCdel, and payload was swapped for pscAAVGFP_888 (Figure 4A, data bar 9). Overall, all the four-plasmid transfections (Figure 4A, data bars 1–9) preserved vg/mL titer performance compared to the three-plasmid transfection (Figure 4A, data bars 10) despite featuring a slightly lower mass of RepCap, helper, and payload plasmids (Table S1).

### Effect on vg/mL yield of combining VP2-TALE8 fusion and TALE-insert payload

The highest measured vg/mL yield of the four-plasmid transfections plotted in Figure 4A featured the combination of pscAAVGFP_888 and RepCap plasmids plus pTALEVP_L9, which encodes the VTL9 fusion protein (Figure 4A, data bar 3, highlighted in green). Due to this high titer, we elected to use pTALEVP_L9 in a set of control transfections (Figure 4B) to assess the influence of combining pTALEVP_L9 and pscAAVGFP_888 within four-plasmid transfection. We performed four-plasmid transfections using helper and pTALEVP_L9 plus either regular pscAAVGFP or pscAAVGFP_888 (888) payload plasmids, and with either regular RepCap plasmid or the RCdel plasmid. We plotted the resulting vg/mL as fold difference from standard, three-plasmid transfection (Figure 4A, purple data bar). For transfections including standard RepCap plasmid, the presence (pscAAVGFP_888) or absence (pscAAVGFP) of the three T8T payload inserts had no statistically significant effect, when comparing the two yields (Figure 4B, data bars 1 and 2). However, when RepCap was swapped for RCdel (Figure 4B, data bars 3 and 4), the absence of T8T payload inserts resulted in a vg/mL yield that was significantly reduced compared to when the T8T payload inserts were present, to a statistically significant degree. We interpreted this observation as being consistent with a hypothesis that the VTL9 fusion protein effects more nuclease protection of progeny viral genomes that possess the three T8T target sequences than standard genomes lacking T8T sequence inserts.

### The vg/mL yield is insensitive to altering VP2-TALE8 fusion plasmid and RepCap plasmid ratio

We next performed transfections with pTALEVP_L9 (Figure 4C) in which helper and pscAAVGFP_888 molar ratio was kept constant (Table S1, transfection types B–E), but the RepCap (or RCdel) plasmid and pTALEVP_L9 molar ratios were varied, within the same overall plasmid mass total (molar ratios indicated in the *x* axis labeling). For VTL9 transfections that included the RepCap plasmid (Figure 4C, gray data bars 1–5), increasing or decreasing the RepCap-to-pTALEVP_L9 plasmid ratio from 1:1 did not significantly change the vg/mL yield performance compared to standard, three-plasmid transfection (Figure 4C purple data bar). For pTALEVP_L9 transfections with the RCdel plasmid (Figure 4C, data bars 6–10), ratio changes also did not significantly change vg/mL yield performance from standard. This preservation of vg/mL yield compared to standard, three-plasmid transfection manifested despite some of the four-plasmid transfections featured in Figure 4C featuring a slightly lower mass of helper, payload, and RepCap or RCdel plasmids (Figure 4C data bars 3–5 and 8–10).

### Impact on infectiousness of four-plasmid transfections with VP2-TALE8 expression plasmids

The presence of all VP2-TALE8 fusion expression plasmids tended to reduce TU/mL (Figure 5A, gray data bars 1–8), compared to the standard, three-plasmid procedure (Figure 5A, purple data bar) and compared to a control three-plasmid transfection in which the RCdel and pscAAVGFP_888 plasmids were used (Figure 5A, data bar 9). This reduction in TU/mL may be due to the slightly lower absolute mass of helper, payload, and RepCap or RCdel plasmids (Table S1) in the four-plasmid transfections or an effect of the presence of the fusion-encoding plasmids, or both factors.

**Figure 5:**
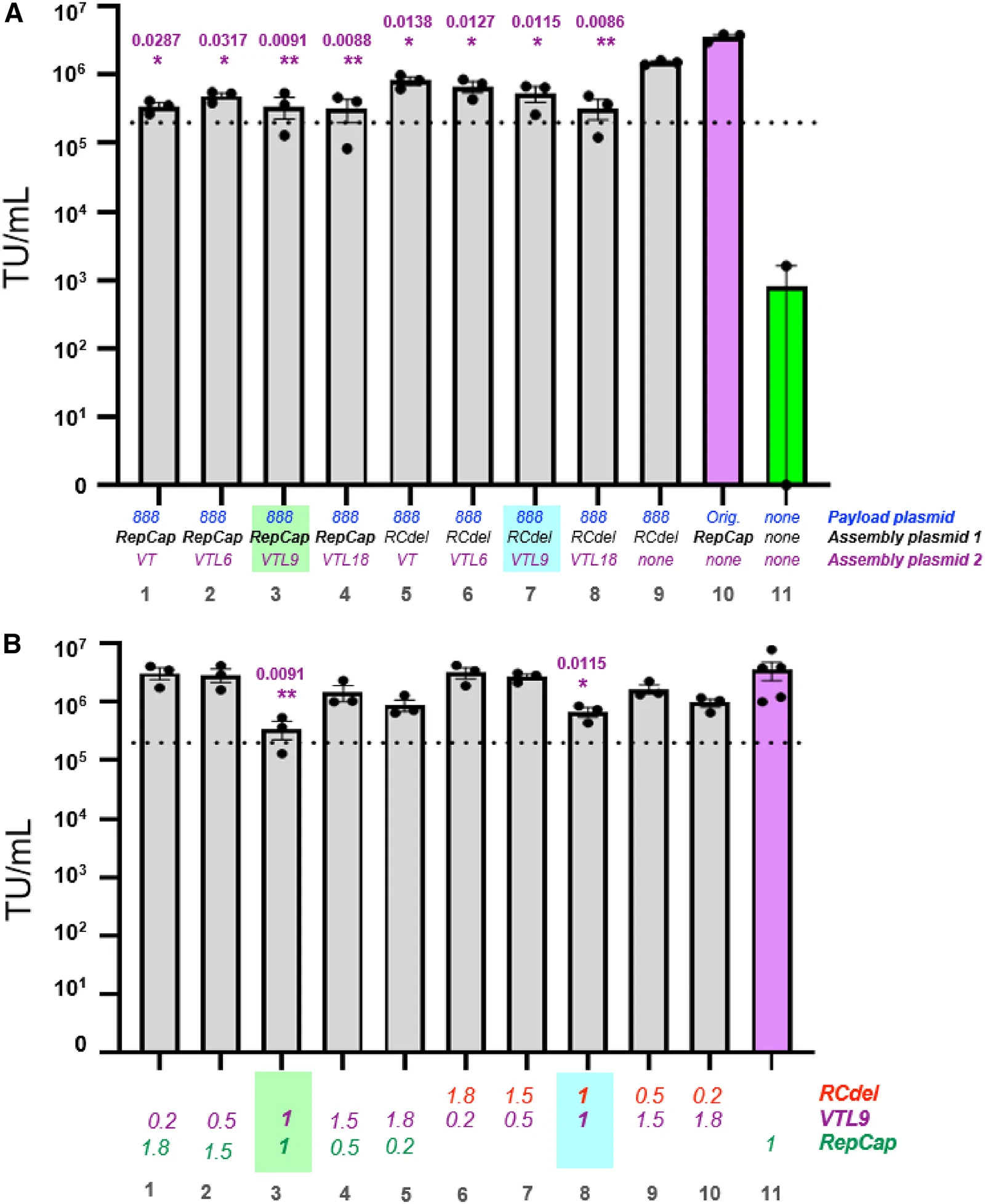
Impact on TU/mL yield of including VP2-TALE8 expression plasmids and T8T payload plasmids within four-plasmid AAV2 production AAV2 production transfections were performed as in Figure 4 and TU/mL yields determined. All data are mean ± SEM of three biological repeats, with determinations made via three flow cytometry measurements per biological repeat. A Brown-Forsythe ANOVA and Dunnett’s T3 post hoc tests was applied to compare a given experimental transfection type (gray bars) to the standard transfection (purple data bar). The TU/mL titers arising from transfected cells (bars 1–9) were all at least two orders of magnitude higher than the none control (green bar). Bars are annotated with a *p* value (purple text) only if a statistically significant difference of *p* < 0.05 (⁎) or *p* = between 0.01 and 0.001 (⁎⁎) arose, and left unannotated if there was no statistically significant difference to comparator. (A) Resulting TU/mL yields from the same transfections as performed in Figure 4A with the LOQ bar in green (HEK293T cells incubated with lysate from untransfected HEK293-F cells. (B) Resulting TU/mL yields from the same transfections as performed in Figure 4C. Values from the plot (A) data bars 3, 7 (highlighted in green and cyan, respectively) are re-plotted in this plot (data bars 3 and 8, respectively).

Due to its vg/mL yield performance in Figure 4C, we again explored different ratios of pTALEVP_L9 within transfections procedures. These data revealed that altering the pTALEVP_L9-to-RepCap plasmid ratio, from 1:1 to 3:1, 9:1, 1:3, or 1:9 (Figure 5B, gray data bars 1, 2, 4, and 5), rescued TU/mL yield back to levels that were not significantly different from that of a standard three-plasmid transfection (Figure 5B, purple data bar). This effect was also observed for transfections featuring RCdel instead of RepCap (Figure 5B, gray data bars 6, 7, 9, and 10). In some cases, this TU/mL yield was rescued despite the four-plasmid transfection featuring lower masses (Table S1) of helper, payload, and RepCap or RCdel plasmids (Figure 5B, data bars 4, 5, 9, and 10) than the standard three-plasmid transfection. While these TU/mL data demonstrate preservation of functional vector production, they do not independently prove an increase in encapsidated genomes.

### Theoretical considerations for VP2-TALE8 fusion co-expression with AAV2 proteins

With respect to AAV2 production transfections, we observed the following in Figures 4 and 5: (1) the presence of pTALEVP_L9 preserved vg/mL yield levels and (2) a DNA sequence specific effect of greater nuclease activity protection when pTALEVP_L9 and pscAAVGFP_888 were both present in transfection.

We next sought to determine the effect of the presence of VP2-TALE8 expression plasmids in four-plasmid transfections on the formation of intact and infection-competent capsids. We considered the following three factors: (1) Soluble, monomeric VP1, VP2, and VP3 proteins associate to form intact capsids (Figure S3A) that are detectable by ELISA. (2) A proportion of the resulting intact capsids are fully active for infection. (3) A proportion of the resulting intact capsids are compromised in some way so as to be non-infectious. The presence of VP2-TALE fusion proteins may influence the proportion of fully active versus compromised capsids either indirectly (Figure S3Bi), without forming part of the resulting capsids, or directly, as a component of the resulting capsids (Figure S3Ci), or via a combination of both interactions. We would anticipate this would also be the case in the absence of wild-type VP2 proteins (Figure S3Bii and S3Cii).

### Impact of VP2-TALE8 expression plasmids on intact capsid formation

Antibodies of the “AAV2 Titer Capsid” commercial ELISA Kit are claimed not to bind VP1, VP2, or VP3 proteins in monomer form or any other configuration not found in native, correctly formed capsids. As such, we used this kit to determine the yield of correctly formed capsids (capsids/mL) arising from three- and four-plasmid transfections. All capsid/mL yields arising from four-plasmid transfections were plotted as fold difference from the capsid/mL yield achieved by standard, three-plasmid transfection (Figure 6A, purple data bar). The presence of expression plasmids encoding VT and VTL6 fusions, plus the RCdel plasmid, and VTL18 fusions plus the RepCap plasmid, showed no significant reduction in capsids/mL compared to standard procedure (Figure 6A, data bars 4, 5, and 6), despite having slightly lower masses (Table S1) of helper, payload, and RCdel plasmids compared to three-plasmid transfections (Figure 6A, data bars 9 and 10). For all other four-plasmid transfections (Figure 6A, data bars 1–3, 7, and 8), the capsid/mL yield tended to be reduced.

**Figure 6:**
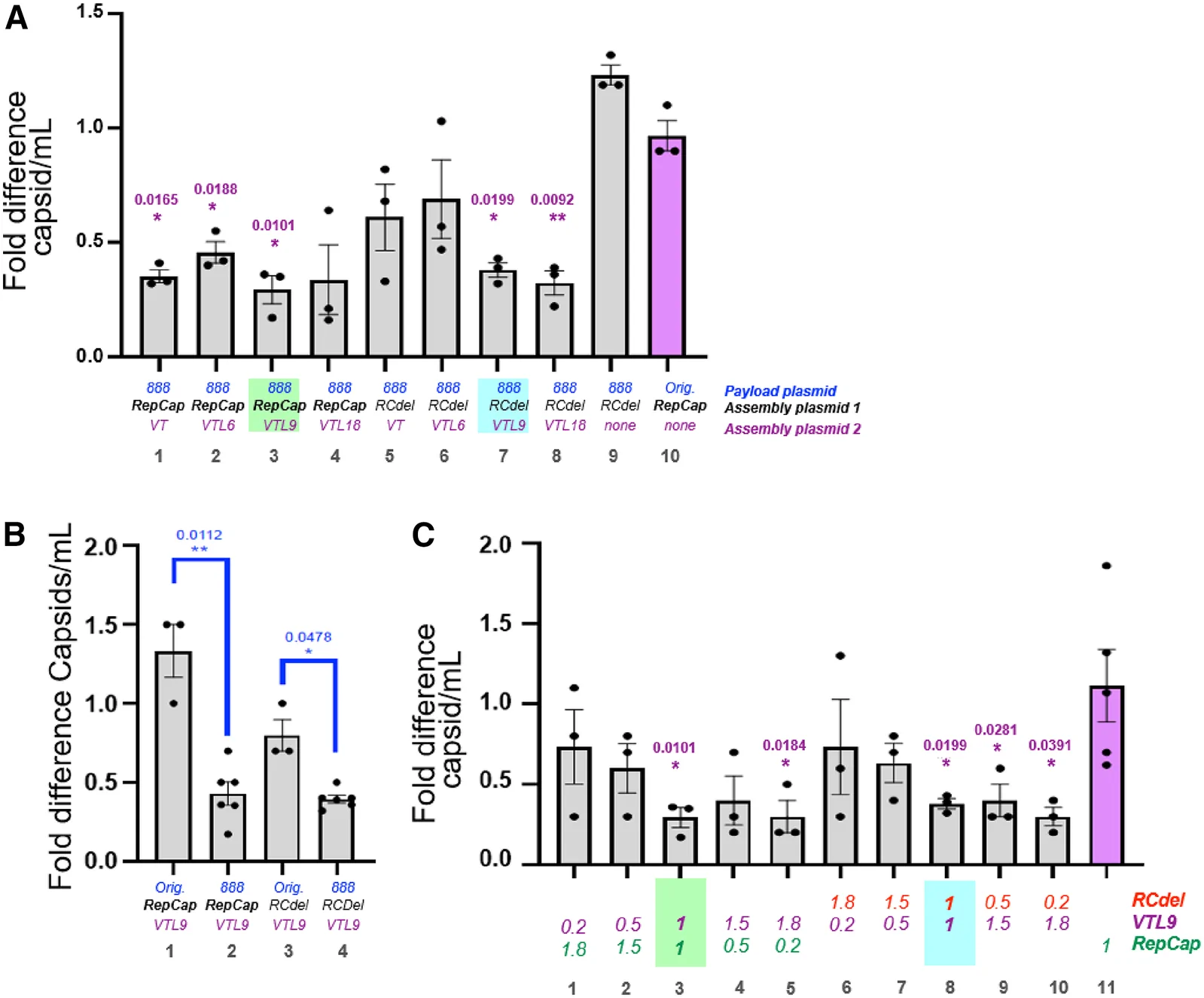
Impact on intact capsid yield of including VP2-TALE8 expression plasmids and T8T payload plasmids within four-plasmid AAV2 production Mean ELISA data ±SEM from the Figure 4 transfections, from at least three biological replicates, with determinations made via two ELISA measurements per biological repeat, were plotted as fold difference between experimental transfection (gray bars) and standard transfection (purple data bar in (A) and (C). For (A) and (C), a Brown-Forsythe ANOVA and Dunnett’s T3 post hoc tests was applied to compare a given experimental transfection type (gray data bars) to the standard transfection data (purple data bar). For (B), a two-tailed Welch’s *t* test was applied to the two pairs of bars indicated by the blue *p* value brackets. Bars are annotated with a *p* value, or *p* value bracket, only if a statistically significant difference of *p* < 0.05 (⁎) or *p* between 0.01 and 0.001 (⁎⁎) arose, and left unannotated if there was no statistically significant difference to comparator. (A) ELISA analysis of the material used in Figure 4A was used to determine intact capsid yields from experimental transfections (gray bars) relative to standard three-plasmid procedure (purple bar). (B) Fold difference between capsids/mL resulting from four-plasmid transfections, using indicated plasmids in addition to helper plasmid, and the capsids/mL resulting from a standard, three-plasmid transfection (A, purple data bar). Bracketed *p* values indicate difference between mean values of the leftmost pair of transfections, and the right most pair. (C) The plot (A) transfections highlighted under the *x* axis in green and cyan were investigated further by altering plasmid ratios as indicated. Values from the plot (A) data bars 3, 7 (highlighted in green and cyan respectively) are re-plotted in plot (C) (data bars 3 and 8, respectively).

We next plotted the capsid/mL yield performance of the same set of two, paired control transfections used in Figure 4B. The presence of the three TALE8 target inserts resulted in a statistically significant drop in capsid/mL yield in the context of a four-plasmid transfection featuring the standard RepCap plasmid, pAAV-RC2, and the VTL9 expression plasmid pTALEVP_L9 (Figure 6B, data bars 1 and 2). This effect was also observed when RepCap was swapped for RCdel (Figure 6B, data bars 3 and 4).

We then determined fold difference in capsid/mL yields arising from transfections with different pTALEVP_L9 plasmid ratios (Figure 6C, gray data bars) compared to standard, three-plasmid transfection (Figure 6C, purple data bar). Reducing the pTALEVP_L9-to-RepCap plasmid ratio, to 1:3 or 1:9 (Figure 6C, data bars 2 and 1, respectively), increased capsid/mL yield back to a level with no statistically significant difference to standard procedure (Figure 6C, purple data bar), despite having been less absolute mass present of payload and helper plasmid in the transfection compared to the standard, three-plasmid transfection method (Table S1; Figure 6C, purple data bar). This was also true when RCdel was included instead of RepCap (Figure 6C, data bars 7 and 6, respectively).

### Impact of the VTL9 expression plasmid and T8T payload insertion on particle morphology and VP proteins present in cell lysate

Figure 3A observations indicated that replacing the RepCap plasmid with a plasmid encoding only a VP2-TALE8 fusion protein abolished TU/mL yield altogether. However, the four-plasmid transfections that included pTALEVP_L9 (encoding the VTL9 fusion protein) and pscAAVGFP_888 (encoding payload with three T8T insertions) were able to rescue TU/mL (Figure 5) performance back to levels comparable with standard, three-plasmid transfection.

We next wished to determine if the use of pTALEVP_L9 and pscAAVGFP_888 in a four-plasmid transfection caused any gross changes to the morphology (Figures 7A–7D) of progeny AAV2 virions detectable by transmission electron microscopy (TEM). Three-plasmid transfections were performed using standard plasmids (Figure 7A) and using RCdel and pscAAVGFP_888 instead of RepCap and pscAAVGFP, respectively (Figure 7B). Four-plasmid transfections were performed using pTALEVP_L9 and pscAAVGFP_888 with helper and RepCap (Figure 7C), and with RCdel instead of RepCap (Figure 7D). For TEM images arising from material from all four transfections, particle diameter was manually determined for 20 spherical structures chosen at random. For all four transfections (Figures 7A–7D), resultant particles had the same gross morphology and were approximately 28 nm diameter, a value typical of AAV particles and their wider viral group, the parvoviruses.^47^ It is important to consider that this preserved gross particle morphology is not evidence of incorporation of VTL9 into viral capsids.

**Figure 7:**
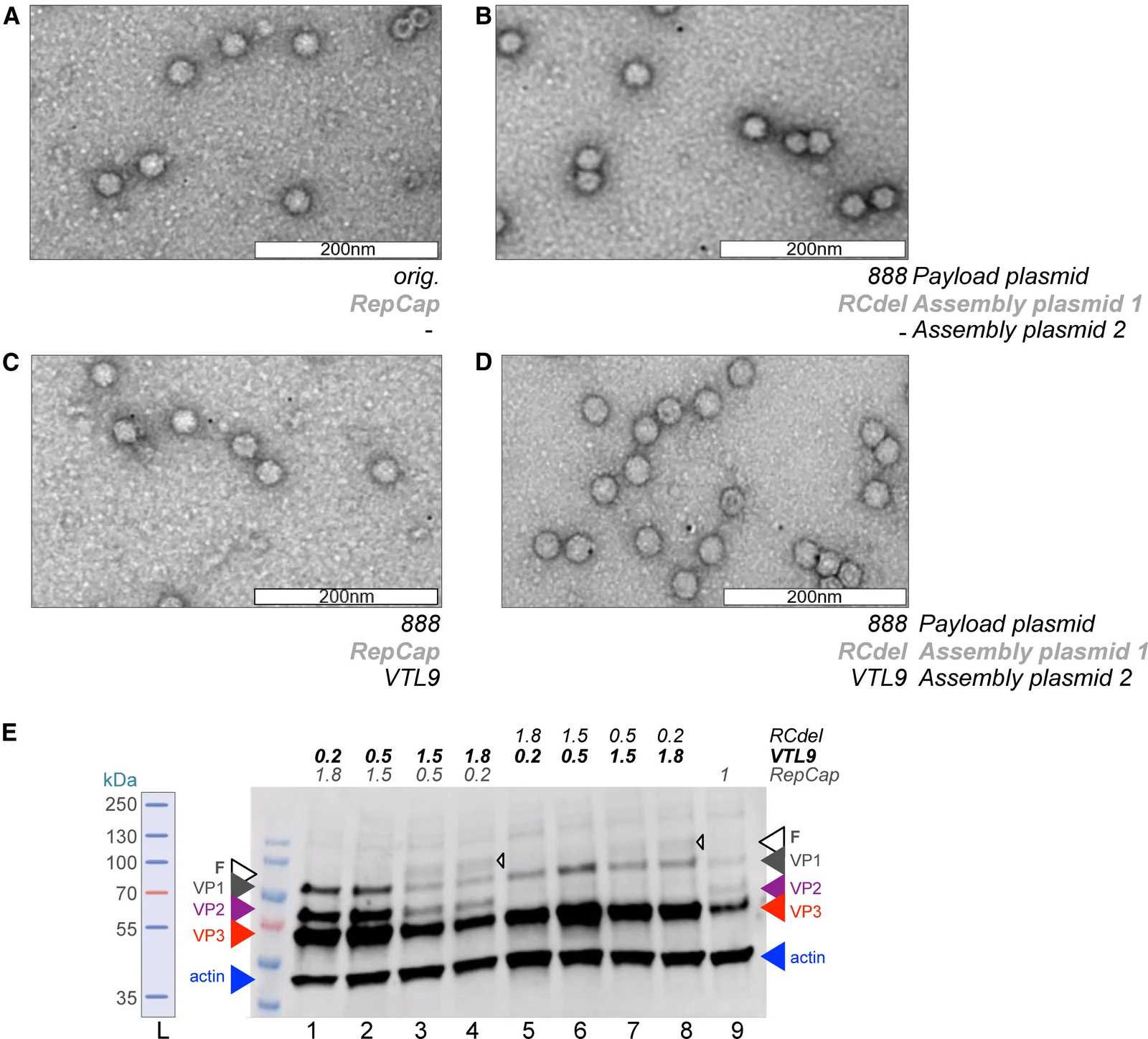
AAV2 particle morphology and host cell lysate VP content (A–D) Transmission electron microscopy (TEM) of AAV2 particles produced using (A) a standard three-plasmid transfection including the original (orig.) payload plasmid and RepCap plasmid, (B) a three-plasmid transfection including the pscAAVGFP_888 payload plasmid (Figure 1D), and the RCdel version of the RepCap plasmid, (C) a four-plasmid transfection including pscAAVGFP_888 and RepCap plasmids plus the plasmid encoding the VTL9 fusion protein, and (D) a four-plasmid transfection including the pscAAVGFP_888, RCdel and VTL9-encoding plasmids. Scale bars, 200 nm. Images are representative of 12–20 images sampled from different sections across a grid at 87,000× magnification for each sample. The black arrow indicates an empty capsid, and the white arrow indicates a full capsid. (E) Western blot analysis of crude lysates arising from four-plasmid transfections with indicated ratios of the plasmid encoding the VTL9 fusion protein and either RepCap or RCdel, probed with the B1 (Progen) antibody that recognized a primary sequence epitope common to VP1, VP2, and VP3, and an anti-β-actin-peroxidase antibody. Color-coded arrowheads indicate the positions of fusion protein (F, white with black outline), VP1 (dark gray), VP2 (purple), VP3 (red), and actin loading control (blue). Numbers 1–9 represent different ratio combinations of plasmid as indicated above the lanes.

We next sought to confirm that gene expression from pTALEVP_L9 resulted in production of protein of the expected size (≈113 kDa) by western blotting (Figure 7E, clearest in lanes 3, 4, 7, and 8). Post-transfection cell lysates were analyzed by bicinchoninic acid assay (BCA) to determine total protein content. All samples were then matched in terms of total protein content before polyacrylamide gel electrophoresis. The gel was then processed for western blotting, with the Merck A3854 anti-β-actin antibody and the Progen Biotechnik B1 antibody. The B1 antibody is described by the manufacturer as recognizing a linear epitope present in VP1, VP2, and VP3 capsid proteins and gives a characteristic staining pattern of a band at >100 kDa for VP1, >70 kDa for VP2, and <70 kDa for VP3.

All samples were positive for the housekeeping protein, β-actin (Figure 7E, lanes 1–9). We interpreted the strength of the β-actin band as an internal loading control, which showed that loading was broadly comparable across samples. For the standard, three-plasmid transfection, using unmodified plasmids only, this procedure yielded a gel lane image with bands corresponding to VP3, VP2, and VP1 at very close to their expected 10:1:1 ratio (Figure 7E, lane 9). All transfections that included the RepCap plasmid showed bands at the expected sizes for VP1, VP2, and VP3 capsid proteins (Figure 7E, lanes 1–4 and 9). For transfections where the RepCap plasmid was swapped for RCdel, the VP2 band was absent (Figure 7E, lanes 5–8), indicating the point mutation^45^^,^^46^ had caused the desired effect. A band at the expected ≈113 kDa size of the VTL9 fusion protein was visible for samples from transfections with a 3:1 or 9:1 ratio of pTALEVP_L9-to-RepCap plasmid (Figure 7E, lanes 3 and 4) but not discernable for the inverse, 1:9 and 1:3 ratios (Figure 7E, lanes 1 and 2). A comparable pattern of VTL9 band visibility was observed for transfections where RepCap was swapped for RCdel (Figure 7E, lanes 7–8), with no VTL9 band discernable for the standard, three plasmid transfection that omitted pTALEVP_L9 (Figure 7E, lane 9).

### Impact of VTL9 expression plasmid and T8T payload insertion on capsid filling as estimated by ELISA and qPCR

ELISA and qPCR data are widely used to infer the level of capsid filling (vg/capsid) in crude AAV lysate.^23^^,^^48^^,^^49^ We used the ELISA data from Figure 6 and the qPCR data from Figure 4 to infer levels of capsid filling achieved for each transfection (Figure 8). The vg/capsid levels achieved by each non-standard (Table S1), four-plasmid transfection (Figure 8A, gray data bars) were plotted as fold difference from that achieved by standard three-plasmid transfection (Figure 8A, purple data bar).

**Figure 8:**
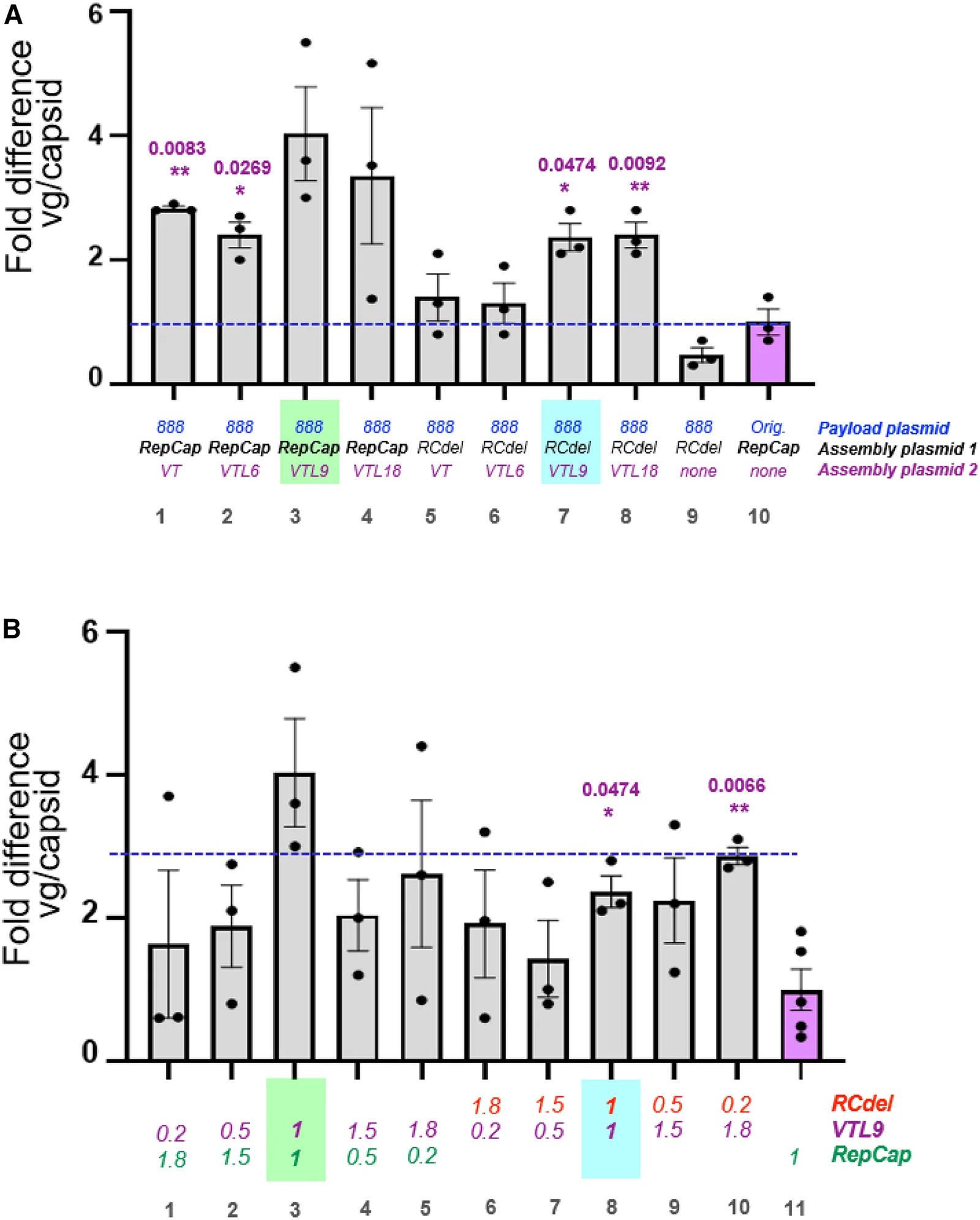
Fold difference in payload filling of AAV2 capsids Payload filling was inferred from mean capsid/mL data (Figure 6) and mean vg/mL data (Figure 4), derived from at least three biological replicates. A Brown-Forsythe ANOVA and Dunnett’s T3 post hoc tests was applied to determine whether there was a statistically significant fold difference between the level of filling (estimated by qPCR and ELISA) arising from a given experimental transfection type (gray data bars) and standard transfection (purple data bars). Each gray data bar is annotated (purple text) with a *p* value only if a statistically significant difference of *p* < 0.05 (⁎) or *p* < 0.01 (⁎⁎) to standard transfection (purple data bar) arose, and left unannotated if there was no statistically significant difference. (A) Fold differences in viral genome-to-capsid ratios (vg/capsid) between transfection with the indicated combinations of plasmids, as used in Figure 4A, and the standard, three-plasmid procedure (set to 1, purple data bar, highlighted by dotted line). (B) Resulting fold difference in vg/capsid between the same transfections as performed in Figure 4C and the standard three-plasmid procedure (purple data bar). Values from the plot (A) data bars 3, 7 (highlighted in green and cyan, respectively) are re-plotted in plot (B) (data bars 3 and 8, respectively).

When VP2-TALE8 fusion expression plasmids were present in transfection, filling levels (as estimated by qPCR and ELISA) compared to standard transfection were either preserved or increased (Figure 8A, gray data bars 1–8) despite featuring a slightly lower mass of RepCap, helper, and payload or RCdel plasmids (Table S1). A control, three-plasmid transfection, with RCdel and pscAAVGFP_888 (Figure 8A, data bar 9), showed no statistically significant change in filling, as determined by qPCR/ELISA-derived estimation. The four-plasmid transfection that included pscAAVGFP_888, pTALEVP_L9, and the RCdel plasmid at a 9:1 molar ratio (Figure 8B, bar 10) showed a significant increase in qPCR/ELISA-estimated filling, approaching 3-fold, while maintaining a comparable level of infectivity (Figure 5B, bar 10). The other transfections, which featured different plasmid ratios of pTALEVP_L9 to RepCap (Figure 8B, bars 1–2 and 4–5) or to RCdel (Figure 8B, bars 6–7 and 9), did not further improve the qPCR/ELISA-estimated filling.

## Discussion

Several of the rational AAV2 capsid fusion designs and transfection regimes tested in this study improved the levels of AAV2 capsid filling as determined by qPCR/ELISA derived estimation, while achieving yield performance across a selection of widely used bioprocess metrics (vg/mL, capsids/mL, and TU/mL) that did not differ from those achieved using standard procedures to a statistically significant extent. The presence of pTALEVP_L9 in four-plasmid transfections did not prevent the formation of viral particles, as determined by TEM, nor did it grossly alter the morphology of progeny virus (Figures 7C and 7D). A band of the size predicted for the VTL9 fusion protein was revealed by western blotting of cell lysate samples only when pTALEVP_L9 had been included in the transfection step (Figure 7E).

### AAV2 production tolerates TALE target insertions in payloads

In the context of standard AAV2 production by three-plasmid transfection, the presence of one, two, or three inserts of the 14 bp T8T sequence in the payload plasmid, pscAAV-GFP, did not significantly compromise production of nuclease-protected viral genomes (Figures 2A and 2B), or the yield of infectious virus (Figures 2C and 2D). The position or number of T8T insertions also did not correlate with the degree of reduction in vg/mL (Figures 2A and 2B) or TU/mL (Figures 2C and 2D) yields. With respect to the MFI signal observed in transduced target cells (Figure S4), no statistically significant difference was detected between the original payload and any of the seven insert-engineered payload variants, although the number of biological replicates (*n* = 2) does not exclude smaller effects that might become detectable with greater replication in subsequent investigations. As such, we would, with caution, suggest the current evidence is consistent with the TALE target insertions not compromising the performance of the payload-encoded expression cassette.

Three T8T insertions represent only an 84 bp (5.3%) increase in the predicted payload size, from 1,578 bp to 1,662 bp, still significantly below the reported 2,400 bp DNA size limit recommended for self-complimentary AAV payloads.^50^ As such, we elected to use the pscAAVGFP_888 plasmid, encoding three T8T insertions, for the rest of the study, to maximize the number of target sequences that might be available for VP2-TALE8 fusion protein binding.

For a clinical application where a large payload needs to be packaged, a possible route to avoid the need for TALE-target insertion would be to design a TALE domain region *de novo*, following the known rules of TALE design,^37^ to target a pre-existing sequence within a therapeutic AAV payload. In this way, no sequence changes to the payload would be necessary.

### TALE-VP2 fusions protect payloads from nuclease activity

Replacing the RepCap plasmid with plasmids encoding only VP2-TALE8 fusion proteins resulted in vg/mL yields that did not differ from those produced by the standard, three-plasmid transfection to a statistically significant extent (Figure 3B). This suggested VP2-TALE8 fusion proteins alone can affect viral genomes in a manner that protects them from nuclease activity. This observation was also consistent with reports that helper plasmid and the host-cell environment can support a level of AAV genome replication^51^^,^^52^ and that exogenous structural proteins engineered to incorporate TALE domains can associate with DNA.^53^

Viral infectiousness, as determined by TU/mL yield, was completely abolished by replacing the RepCap plasmid with plasmids encoding only VP2-TALE8 fusion proteins (Figure 3A). This may be due to the absence of VP1, and as such the VP1 phospholipase domain which is understood to be essential for viral escape from target cell endosomes.^54^ Alternatively, this may also be due to more profound structural compromises caused by the absence of native VP and Rep proteins. Such non-native capsid structures have not been directly reported for AAV but have been explored *in silico* for icosahedral viral particles in general.^55^^,^^56^

We next trialed a set of four-plasmid transfections, each featuring standard RepCap and helper plasmids but with pscAAVGFP_888 instead of regular payload and a plasmid encoding a VP2-TALE fusions as the fourth, additional plasmid (Figure 4). Four-plasmid transfections that included pTALEVP_L9, encoding the VTL9 fusion protein with specific ratios (Figure 4A, data bars 3 and 7), maintained a comparable level of vg/mL yields to standard, three-plasmid AAV2 production (Figure 4A, data bar 10). It is important to also note that Figure 4 observations cannot differentiate between the following scenarios in which VTL9 protects AAV payloads: (1) encapsidated AAV payloads interact with VTL9 as an incorporated capsid component, (2) non-encapsidated AAV payloads interact with free VTL9 that is not a part of intact capsids, (3) via indirect effects or (4) via a combination of these scenarios.

### Protection from nuclease by a VP2-TALE fusion is partly payload sequence-dependent

It might be anticipated that the vg/mL yield performance from three-plasmid transfections in Figure 3B would be impacted by using the pscAAV-GFP_888 instead of the unmodified pscAAV-GFP payload plasmid, given the expectation of enhanced capsid:payload association via TALE8-to-T8T binding. However, swapping pscAAV-GFP_888 for pscAAV-GFP had no clear impact on vg/mL yield in that case. To investigate this further, we performed four-plasmid transfections including pTALEVP_L9 and RepCap plus either regular pscAAVGFP or pscAAVGFP_888 payload plasmids (Figure 4B, data bars 1 and 2). The presence of the T8T inserts had no significant effect. However, using the RCdel plasmid, which encodes no VP2 expression, revealed a sequence-specific effect, with the absence of T8T inserts resulting in a significantly lower level of production of nuclease-protected payload genomes (Figure 4B, data bars 3 and 4).

We suggest a sequence-specific effect is manifested by the presence of the VTL9 fusion protein, encoded by pTALEVP_L9, which is partly masked by the beneficial presence of wild-type VP2 in transfections that feature equimolar amounts of pTALEVP_L9 and RepCap. However, it is important to note that the Figure 4B observation does not prove any direct interaction between VTL9 and T8T target sequences. Further experimentation, beyond the scope of this current work, will be needed to elucidate the mechanism driving these differences in vg/mL yield.

### Intact capsid formation in the presence of VP2-TALE fusion is payload sequence-dependent

Intact capsid formation (capsid/mL by ELISA) was reduced only when a VP2-TALE fusion protein and payload with TALE target inserts were present (Figure 6B). This may be an indirect (Figure S3B) or direct (Figure S3C) effect. The sequence-dependent observation tempts us to speculate that targeted binding to payload genomes by the fused TALE domain may be taking place. Such DNA-bound capsid components may hinder intact capsid formation via steric obstruction. However, these differences in capsid/mL yield do not directly evidence VTL9:T8T binding. Further mechanistic studies will be needed in future to characterize these effects.

### VP2-TALE fusion plus TALE-targets within the payload combine to increase capsid filling, as determined by qPCR/ELISA-derived estimation

Combining data from Figures 4 and 6 also allowed us to infer the level of capsid filling achieved by different transfections (Figure 8). Four-plasmid transfections that included plasmids encoding a VP2-TALE fusions generally had higher levels of capsid filling than standard, three-plasmid AAV2 production, with the presence of pTALEVP_L9 being associated with the highest increase in filling, of more than 2-fold (Figure 8A). Altering the pTALEVP_L9/RepCap plasmid mass ratio within this transfection setup did not improve upon this filling level increase (Figure 8B). However, altering the pTALEVP_L9-to-RepCap plasmid molar ratio within a four-plasmid transfection could increase TU/mL (Figure 5B) and capsid/mL (Figure 6C) yields back to levels comparable with standard, three-plasmid AAV2 production. For instance, the approaching 200% increase in capsid filling (as estimated by qPCR and ELISA) observed when combining the VP2-negative RCdel RepCap variant, the VTL9 expression plasmid, helper, and 888 payload plasmids (Figure 8B, data bar 10), showed no statistically significant reduction in TU/mL yield (Figure 5B, data bar 10) compared to standard transfection.

Within the four-plasmid transfections overall, those that included pTALEVP_L9 or pTALEVP_L18, encoding the VTL9 and VTL18 fusion proteins, respectively, exhibited higher levels of filling (as estimated by qPCR and ELISA) than those that included pTALEVP_01 or pTALEVP_L6, encoding VT and VTL6 (Figure 8A). VTL9 and VTL18 fusion proteins have longer linkers and slightly higher serine residue content (26.47% and 30%, respectively) compared to VTL6 (25%) and linkerless VT (Figure 1B). Rep proteins must associate with payload DNA in order to mediate its threading into the 5-fold symmetry channel (Figure S1) of assembled capsid particles.^57^ The relationship observed in Figure 8A between VT fusion protein linker type and capsid filling improvement hints that the accessibility of the TALE8 domain may influence its ability to bind payload in a manner that is compatible with Rep protein interactions.

All transfections performed in this study used 1.5 μg total plasmid DNA (Table S1), and the identity of the plasmids used in each transfection clearly was a strong influencing factor the results obtained. However, we did not match the absolute mass of payload, helper, and RepCap or RCdel plasmids across three-plasmid and four-plasmid transfections. As such, we cannot rule out transfection efficiency effects that could arise from the different mass proportions of the different plasmids present, as a contributing factor to the differences between three-plasmid and four-plasmid data.

One solution to this limitation would be to match the mass of each plasmid common to the three-plasmid and four-plasmid transfections, with the total DNA mass of the three-plasmid transfections subsequently equalized using an unrelated “makeweight” plasmid or non-coding DNA. However, such an approach would introduce a different potential confounding variable. Nominally non-functional carrier, filler, or empty-vector DNA has itself been reported to alter transfection efficiency and transgene expression, with effects dependent upon vector identity, DNA topology, cell type, and transfection system.^58^^,^^59^^,^^60^ For example, empty expression vectors can either stimulate or inhibit co-transfected transgene expression,^58^ while non-specific filler DNA can increase the proportion of transfected cells and steady-state transgene mRNA levels.^59^ Thus, although the use of a makeweight plasmid would control total DNA mass and the absolute quantities of the experimental plasmids, it could not necessarily be assumed to be biologically or transfectionally inert and could therefore introduce an alternative source of experimental confounding.

### Conclusions

This study identified several rational AAV2 capsid fusion designs and transfection regimes that improved the levels of AAV2 capsid filling while preserving yield performance across a selection of widely used bioprocess metrics (vg/mL, capsids/mL, and TU/mL). Future studies, beyond the scope of this current work, will be needed to evidence the underlying mechanistic causes of the bioprocess benefits we have reported here. For instance, data reported here do not rule out the possibility that the VP2-TALE8 fusion proteins associate with payload genome DNA in a manner that is not sequence specific or that they fail to be incorporated into progeny viral capsids. Our observations also do not exclude the possibility that the fused TALE8 domains act only to increase the frequency of binding events between capsids, Rep proteins, and payload DNA in an entirely non-specific manner that could also be achieved by many different protein domains. Direct, *in vitro* experiments, such as DNA band shift or viral particle capture using immobilized DNA ligands, may provide a clearer picture. Further structural interrogation of particles using methods such as mass spectrometry and cryo-EM will also provide a clearer picture regarding interactions and stoichiometry of capsid components and payload genomes.

To our knowledge, prior to this work, the largest domain fused to VP2 and compatible with transfection-based AAV2 production was the ≈32 kDa *Mycobacterium tuberculosis* Ag85A antigen.^61^ As such the 46–48 kDa (including linker) TALE8 domains reported here represent a new threshold in terms of the size of protein fused to the VP2 N terminus. The compatibility we have demonstrated between AAV2 production and VP2 fusion domains of this size may be of value beyond the bioprocess engineering community, for clinical researchers seeking to use capsid fusions, for improved AAV2 targeting,^61^^,^^62^ or to build entirely novel, protein-mediated therapeutic capabilities into capsid structures.

In conclusion, our systematic engineering of VP2-TALE8 fusion proteins and complementary modifications to the payload genome have yielded a novel AAV2 production system with substantially improved capsid filling efficiency, as estimated by qPCR and ELISA. As capsid filling was determined using indirect, estimation-based methods orthogonal, particle-level analyses will be required to definitively confirm the magnitude of the capsid-filling effects reported in this current work. Our capsid-engineering approach addressed a critical limitation in current AAV2 vector manufacturing and could contribute to the development of more effective and safer gene therapies. The compatibility of our system with standard production methods suggests potential for straightforward implementation in existing manufacturing processes, offering a practical path to improved AAV2 vector production.

## Materials and methods

### Plasmid design and propagation

The helper plasmid pAdDeltaF6 (Addgene plasmid #112867) was a gift from James M. Wilson, and the payload plasmid pscAAV-GFP (Addgene plasmid #32396) was a gift from John T. Gray. The AAV2 RepCap genes were encoded by plasmid pAAV-RC2 (Cell Biolabs VPK-422). All other plasmids were generated by gene synthesis and assembly procedures performed by Genscript. Plasmids pTALEVP_01, pTALEVP_L6, pTALEVP_L9, and pTALEVP_L18 were designed to encode VT, VTL6, VTL9, and VTL18 fusion proteins, respectively (Figure 1C). Complete sequence chromatographic data for all fusion protein expression plasmids and pscAAV-GFP payload variant plasmids are provided in the Chen et al. (2026)^63^ online dataset. Complete sequence data for the RCdel plasmid is provided in the Bal et al. (2026)^64^ online dataset. All fusion protein expression cassettes consist of a CMV promoter followed by ORF followed by bovine growth factor polyA signal. Standard molecular biology techniques were also used for plasmid propagation, isolation, and analytical procedures.

### Mammalian cell cultivation

Cells of the “FreeStyle HEK293-F” cell line (Life Technologies Limited, Paisley, UK, cat. no. R79007) were cultured in FreeStyle 293 Expression Medium (Life Technologies, cat. no. 12338018) under conditions of 37°C, 5% CO_2_, and 135 rpm shaking in a humidified incubator (PHC Europe B.V., Loughborough, UK, MCO-170AICD-PE). The cells were passaged every 3–4 days to maintain viability above 90% and over a density range of 2 × 10^5^–3 × 10^6^ cells/mL in 20 mL of culture. HEK293T adherent cells, sourced from the American Type Culture Collection (ATCC, cat. no. CRL-3216), were propagated in Dulbecco’s Modified Eagle Medium (DMEM; Gibco, UK), supplemented with 10% v/v heat-inactivated (Gibco, UK) fetal bovine serum (FBS). HEK293T cells were cultured in a humidified incubator (PHC Europe B.V., Loughborough, UK, MCO-170AICD-PE) under conditions of 37°C and 5% CO_2_.

### Transient transfections for AAV2 production

24 h prior to transfection, suspension HEK293-F cells were seeded to give a total volume of 25 mL media with a cell density of 5 ×10^5^ cells/mL in a 125 mL Erlenmeyer flask (Corning, cat no. 431143). At transfection 1 mL aliquots of the cell solution were transferred to individual wells of six-well plates (Sarstedt, Germany, 83.3920.500). For both three-plasmid and four-plasmid transfections, 1.5 μg total plasmid DNA was used per well. A 1:1, μL:μg, ratio of PEIpro (Sartorius Stedim, Epsom, UK, 101000017) reagent volume to DNA mass was applied for all transfections. For three-plasmid transfections a 1:1:1 molar ratio was always used for the helper, RepCap, and payload plasmids (Figure 1F). This mass and molar ratio was also used when pscAAV-GFP was swapped for a payload variant with T8T inserts and when the RepCap plasmid was substituted either for plasmids encoding VP2-TALE8 fusions proteins or for the RCdel variant of the RepCap plasmid.

Unless otherwise stated, all four-plasmid transfections were performed with a 1:1:1:1 molar ratio of helper, payload (or engineered payload), RepCap (or RCDel variant) and plasmid encoding VP2-TALE8 fusion protein. Where indicated, some four-plasmid transfections feature those plasmids, in order, but with the final two plasmids having a 0.2:1.8 molar ratio (and the reverse) or a 0.5:1.5 molar ratio (and the reverse).

For each given transfection, Opti-MEM (Fisher Scientific, Loughborough, UK, cat. no. 12559099) was added to plasmid DNA to a total volume that represented 5% of the total cell culture volume and, in a separate vessel, Opti-MEM was added to PEIpro also to a final volume equal to 5% of the total, final cell culture volume. These PEIpro and DNA mixtures were then incubated for 15 min at room temperature (RT). The combined transfection complexes, now constituting 10% of final total cell culture volume, were added dropwise to the cell culture.

### AAV2 harvest

72 h after addition of the PEI:DNA complexes, a volume of Gibco AAV-MAX Lysis buffer (Fisher Scientific, Loughborough, UK, cat no. 17331899) equivalent to 10% of the total cell culture volume was added to each well of transfected cells before returning them to the incubator. Two hours later the resulting crude lysate material from each well was transferred to Eppendorf tubes, followed by centrifugation at 12,000 rpm for 10 min in a Sigma 1–14 centrifuge. Supernatant was then removed and distributed across several aliquots which were immediately stored at −80°C.

### AAV2 genome titration by qPCR

Real-time qPCR was performed to quantify AAV2 vg/mL present in crude lysate. As a standard procedure intended to eliminate non-encapsulated DNA prior to qPCR, 5 μL of the sample was diluted with 35 μL of molecular biology-grade water (Corning, UK; 46–000-CM), 5 μL of 10X DNase I reaction buffer (New England Biolabs, Hitchin, UK, M0303S), and 5 μL of 2 U/μL DNase I (New England Biolabs, UK, M0303S), followed by incubation at 37°C for 30 min. These samples were then heated at 95°C for 10 min to denature capsids and release the encapsulated payloads. A primer pair targeting the GFP ORF within the genomic payload, GTCCGCCCTGAGCAAAGA and TCCAGCAGGACCATGTGATC, was used for amplification.^65^ Serially diluted samples were mixed with the SsoAdvanced Universal SYBR Green Supermix (Bio-Rad, Watford, UK, 1725271) and loaded onto the CFx Connect device (Bio-Rad, UK). A standard curve was generated using serial dilutions of a quantity of linearized pscAAVGFP plasmid of known purity and concentration, to correlate cycle threshold (Ct) values with genome quantity. The qPCR thermal cycling program was set up according to the SsoAdvanced Universal SYBR Green Supermix’s manual. Briefly, this template-containing samples were briefly incubated at 98°C to activate the proprietary polymerase, followed by a three-minute initial DNA denaturation step then 39 cycles of a 15-s, 98°C denaturation step and a 30-s, 60°C annealing and extension step.

### Western blotting

All western blot procedures were performed at room temperature unless otherwise stated. Total proteins content of cell lysate samples was quantified using the Pierce BCA Protein Assay Kit (Thermo Scientific, UK, 23225). Samples were then diluted in PBS (Thermo Scientific, UK, 15424389) as required to give all samples a total volume of 30 μL and the same total protein concentration. 9 μL of Laemmli buffer (Bio-Rad, Watford, UK, cat. no. 1610747) and 1 μL of 10% 2-mercaptoethanol (Bio-Rad, cat. no. 1610710) was then added to the sample before incubation at 95°C for 5 min. Samples were then run on 10% Mini-PROTEAN TGX Protein Gels (Bio-Rad, cat. no. 4568034) and subsequently transferred to nitrocellulose membranes using Trans-Blot Turbo Mini 0.2 μm nitrocellulose transfer packs (Bio-Rad, cat. no. 1704158). Membranes were blocked with EveryBlot Blocking Buffer (Bio-Rad, cat. no. 12010020) for 5 min. The membrane was first incubated with the anti-AAV VP1/VP2/VP3 mouse monoclonal antibody (Progen Biotechnik GmbH, Heidelberg, Germany, cat. no. 690058) at 1/250 dilution for 1.5 h, followed by incubation with the horseradish peroxidase (HRP) conjugated Rabbit Anti-Mouse IgG H&L antibody (Abcam, Cambridge, UK, cat. no. AB6728-1001) at 1/2,000 dilution for 1 h. The membrane was then further incubated with the anti-β-actin-peroxidase antibody (Merck, cat. no. A3854) at 1/10,000 dilution for 1.5 h. Five five-minute washes with Tris-buffered saline (Bio-Rad, cat. no. 1706435) and 0.05% v/v Tween 20 (Merck Life Science cat. no. P9416) were performed between antibody incubations. Protein detection was performed using Clarity Max Western ECL substrate (Bio-Rad cat. no. 1705062), and luminescent signals were visualized with the Amersham Imager 600 (Cytiva).

### ELISA

Cell lysate samples were analyzed using the AAV2 Titer Capsid ELISA Kit (GenScript, Oxford, UK, cat. no. L00942) according to the manufacturer’s instructions. Briefly, samples were serially diluted and loaded onto a pre-coated plate followed by addition of biotinylated anti-AAV2 antibody and streptavidin-HRP, with washing steps between each incubation. Next, a solution of the HRP substrate, 3,3′,5,5′-tetramethylbenzidine, was added followed by the stop solution. The color intensity in each well was measured at 450 and 650 nm using the CLARIOstar plate reader (BMG LABTECH, Aylesbury, UK). To correct for background signal due to turbidity, the absorbance at 650 nm was subtracted from the absorbance at 450 nm for each well. A standard curve was generated using a four-parameter logistic (4-PL) model, with known concentration of AAV2 control material provided in the kit with concentration (capsids/mL) on the *x* axis and the corresponding mean absorbance value on the *y* axis, using GraphPad Prism v.9.4.1.

### Transduction procedure and flow cytometry

Adherent HEK293T target cells were seeded at 1 × 10^6^ cells/mL with 10% v/v FBS DMEM into wells (5 × 10^4^ cells/well) of a 96-well plate (Fisher Scientific, Loughborough, UK, 10212811) or seeded at 1 × 10^5^ cells/well with 600 μL cell culture volume each well in a 12-well plate (Falcon, 353043) before transfer to an incubator 1 h prior to addition of cell lysates from AAV2 production. 15 μL of cell lysate samples were serial diluted with 10% v/v FBS DMEM, with 12 μL of each diluted sample added to each well. For a target cell transduction volume of 600 μL, 100 μL of cell lysate samples was diluted 1:2 with 10% v/v FBS DMEM, with 100 μL of diluted sample added to each well. 24 h after cell lysate sample addition, for transduction in 96-well plate, each well was topped up with 100 μL of fresh 10% v/v FBS DMEM. For transduction in 12-well plate, each well was topped up with 300 μL of fresh 10% v/v FBS DMEM.

At 72 h after cell lysate sample addition, target cells were trypsinized (Gibco, 25200056) and fixed by addition of 4% v/v paraformaldehyde PBS (Thermo Scientific, UK, 15424389) before being placed at room temperature for 10 min. The plate was centrifuged at 1,000 rpm (Eppendorf, 5910R). Supernatant was removed using a multichannel pipette (Starlab, S7108-3300) and cell pellets were resuspended in PBS and transferred to a round bottom 96-well plate (Thermo Scientific, 268200) prior to flow cytometry.

Samples were analyzed using an LSRFortessa™ Cell Analyzer (BD Biosciences, UK) with excitation at 488 nm. A minimum of 10,000 cell measurement events were recorded per sample and used for analysis. Data gathered from flow cytometry experiments were analyzed using Fojo v.10.7.1 (BD Biosciences, UK) software. Forward and side scatter gating were applied to isolate cells, and forward scatter height and forward scatter area were applied to isolate singlet cells. The percentage of GFP-positive cells was quantified and normalized against non-transduced cells. The number of transducing units per mL was calculated using the following formula:

(Equation 1) $$\frac{\text{TU}}{\text{mL}}=\frac{\%\;\text{GFP}\;\text{positive}\;\text{cells}*\text{number}\;\text{of}\;\text{cells}\;\text{at}\;\text{transduction}}{\text{vector}\;\text{input}\;\text{volume}}\;*\text{Dilution}\;\text{factor}$$

### TEM

For sample preparation for TEM, the AAV2 transfection procedure described previously was proportionally scaled to 25 mL in 125 mL Erlenmeyer shake flasks (Coring, 431143) flasks. After the 72-h production period, the cell culture was centrifuged at 2,000 rpm (Eppendorf, 5910R) for 15 mins and then resuspended in 1 mL PBS. A volume of Gibco AAV-MAX Lysis buffer equivalent to 10% of the cell suspension was added, which was then incubated at 135 rpm for 2 h, followed by three cycles of freezing and thawing in a dry ice with 70% v/v ethanol bath and a 37°C water bath. 0.8 μL (200 units) of Benzonase nuclease (Merck cat. no. E1014) was added to each sample and incubated at 37°C for 1 h. Cell lysates were then centrifuged at 12,000 rpm for 10 min (Sigma 1–14) and the supernatant transferred to an Eppendorf tube and stored at 4°C prior to purification.

1 mL of the supernatant from each sample was purified using Dynabeads CaptureSelect AAV Magnetic Beads (Life Technologies, cat. no. 2853522001). Briefly, samples were incubated with beads for 30 min in Eppendorf tubes and with rotation in a tube rotator (Cole-Parmer, TR-200). Sample tubes were then placed in an Invitrogen DynaMag-2 Magnet (Fisher Scientific, 10723874) to immobilize beads. Two washes were then performed, using 500 μL of PBS. Samples were eluted from the Dynabeads CaptureSelect AAV Magnetic Beads with 60 μL of 50mM citric acid, pH 2.5–3.0 and neutralized with 1 μL of example, 1 M Tris, pH 8. Formvar/Carbon films on 200-mesh Cu grids (TAAB, Berkshire, UK, cat. no. F077/100) were glow discharged using the PELCO easiGlow machine (Ted Pella, Inc, United States, California). A 3 μL aliquot of purified sample was applied to the grid and incubated at RT for 2 min. Excess sample was carefully removed, and 3 μL of 1% uranyl acetate was added to the grid, followed by a 40-s incubation at RT. The grids were then dried for approximately 20 min before imaging with an FEI Tecnai T12 transmission electron microscope device (Thermo Fisher).

## Data and code availability

Sequence data for the VP2-TALE fusion protein expression plasmids and AAV payload variant plasmids bearing TALE-binding target sequence insertions are available at the Figshare dataset links cited and provided in the text. All other data generated or analyzed during this study (qPCR, ELISA, flow cytometry, western blot, and TEM data) are available from the corresponding author upon reasonable request.

## Acknowledgments

We gratefully acknowledge the support of the 10.13039/501100000266Engineering and Physical Sciences Research Council via grant number EP/X025446/1, and Cytiva. We thank Jemima Burden and Ian White of the Laboratory for Molecular Cell Biology at University College London for TEM instruction and support.

## Author contributions

Conceptualization, Q.C., R.W., and D.N.N.; methodology, Q.C., M.B., G.H., R.W., and D.N.N.; investigation, Q.C., R.W., and D.N.N.; visualization, Q.C., R.W., and D.N.N.; supervision, Q.C., R.W., and D.N.N.; writing – original draft, Q.C., R.W., and D.N.N.; writing – review and editing, Q.C., R.W., and D.N.N.

## Declaration of interests

The authors declare that they have no known competing financial interests or personal relationships that could have appeared to influence the work reported in this paper.

## Supplemental information

Document S1. Figures S1–S4 and Table S1

Document S2. Article plus supplemental information
