# Supplementary material for: VP2-TALE fusions increase AAV2 capsid filling with payload bearing cognate TALE target sequences as determined by qPCR/ELISA
Source: Mol Ther Adv. 2026 Oct 26;34(4):201877. doi: 10.1016/j.omta.2026.201877 (PMC13770668; doi:10.1016/j.omta.2026.201877)
Supplement: Supplementary file 1 [file mmc1.pdf]

OMTA, Volume *34*

Supplemental information

VP2-TALE fusions increase AAV2 capsid filling with payload bearing cognate TALE target sequences as determined by qPCR/ELISA

Qiantong Chen, Mehtap Bal, Geoffrey Howe, Robert Whitfield, and Darren N. Nesbeth

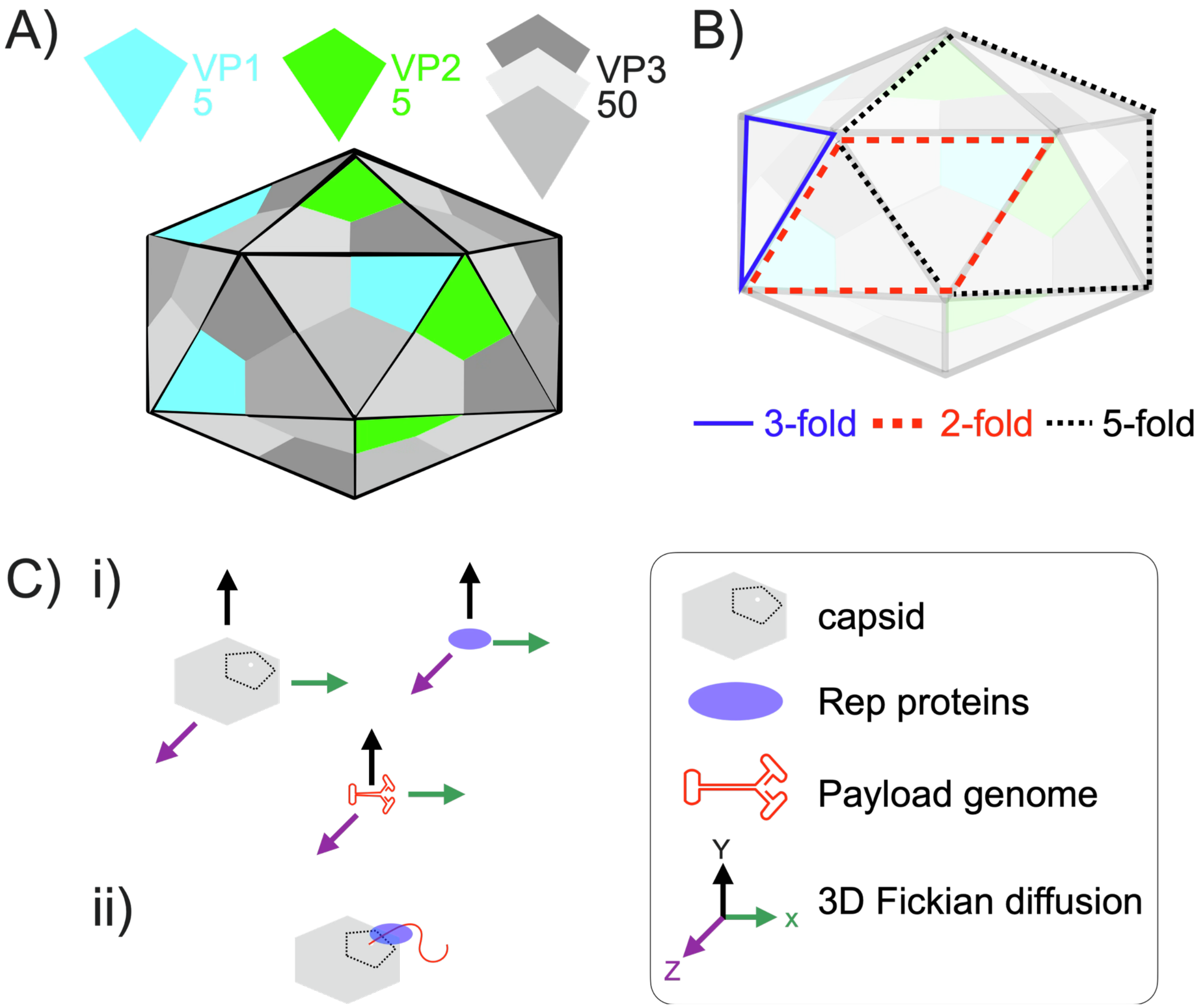

Figure S1. Structure and assembly concepts for AAV2 capsids. A) Schematic representation of AAV2 capsid composition showing each of the three capsid proteins, VP1 (cyan, 5 copies), VP2 (green, 5 copies), and VP3 (contrasting shades of grey, 50 copies), as irregular quadrilaterals arranged in an icosahedral structure. **B)** The icosahedral structure of AAV2 capsid with lines to highlight capsid regions associated with the 3-fold (blue), 2-fold (red), and 5-fold (black) symmetry axes. **C)** Proposed model of AAV2 capsid assembly and genome packaging: **i)** Free capsid proteins, Rep proteins, and the payload genome undergo three-dimensional (3D) Fickian diffusion within host cell interior; **ii)** Rep proteins facilitate the interaction between the capsid and payload genome for packaging.

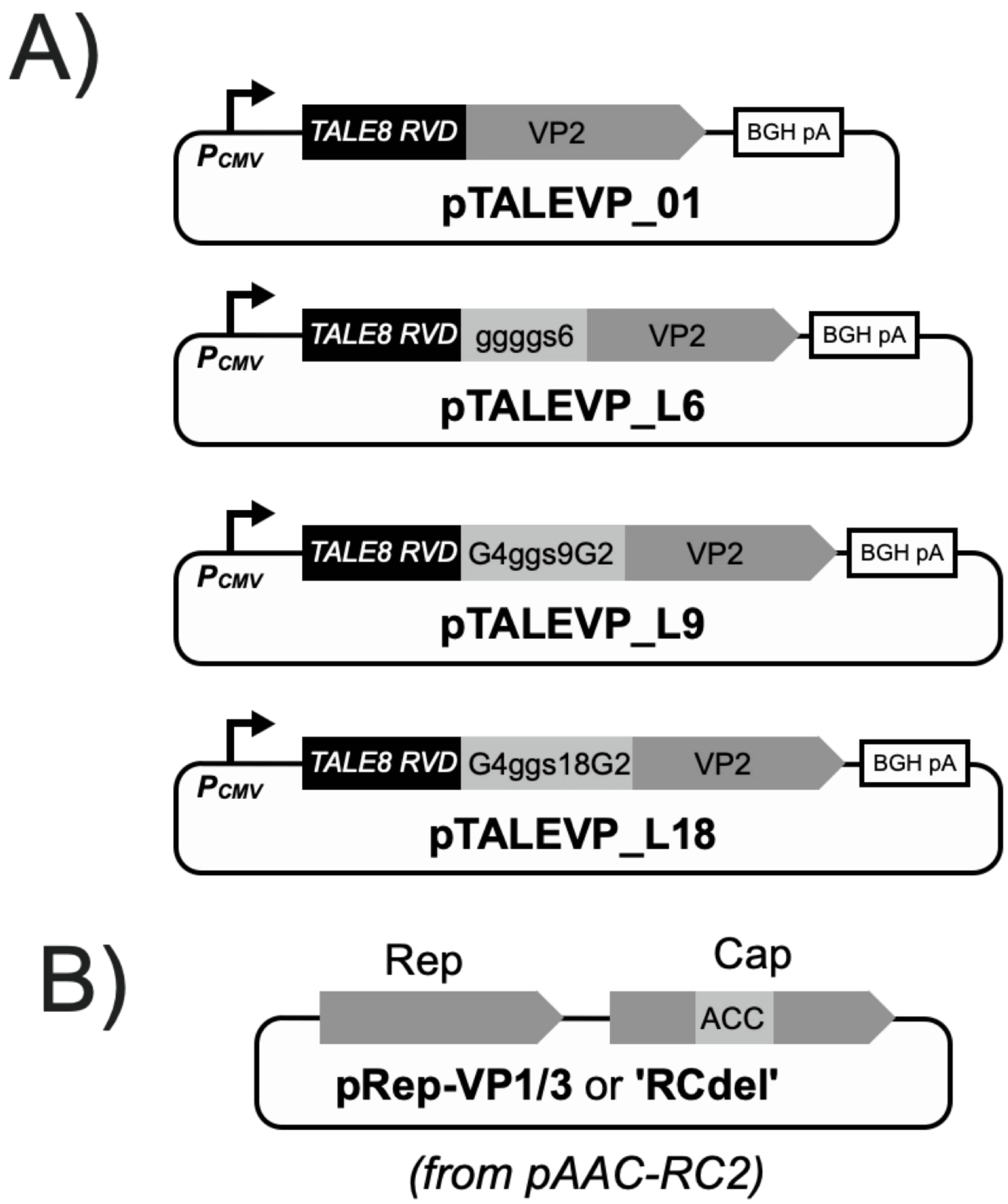

Figure S2. Summary diagrams of plasmids engineered for use in this study. A) Fusion protein expression plasmids all featured the same cytomegalovirus (CMV) promoter and polyadenylation (pA) signal from bovine growth hormone (BGH) gene. Assembly and sequence details for these four plasmids can be found in the Chen et al. (2026)[64] Figshare data deposit. **B)** The pAAV-RC2 RepCap plasmid was modified via single C-to-G base-pair substitution to generate pRep-VP1/3. Assembly and sequence details available in the Bal et al. (2026)[65] Figshare data deposit.

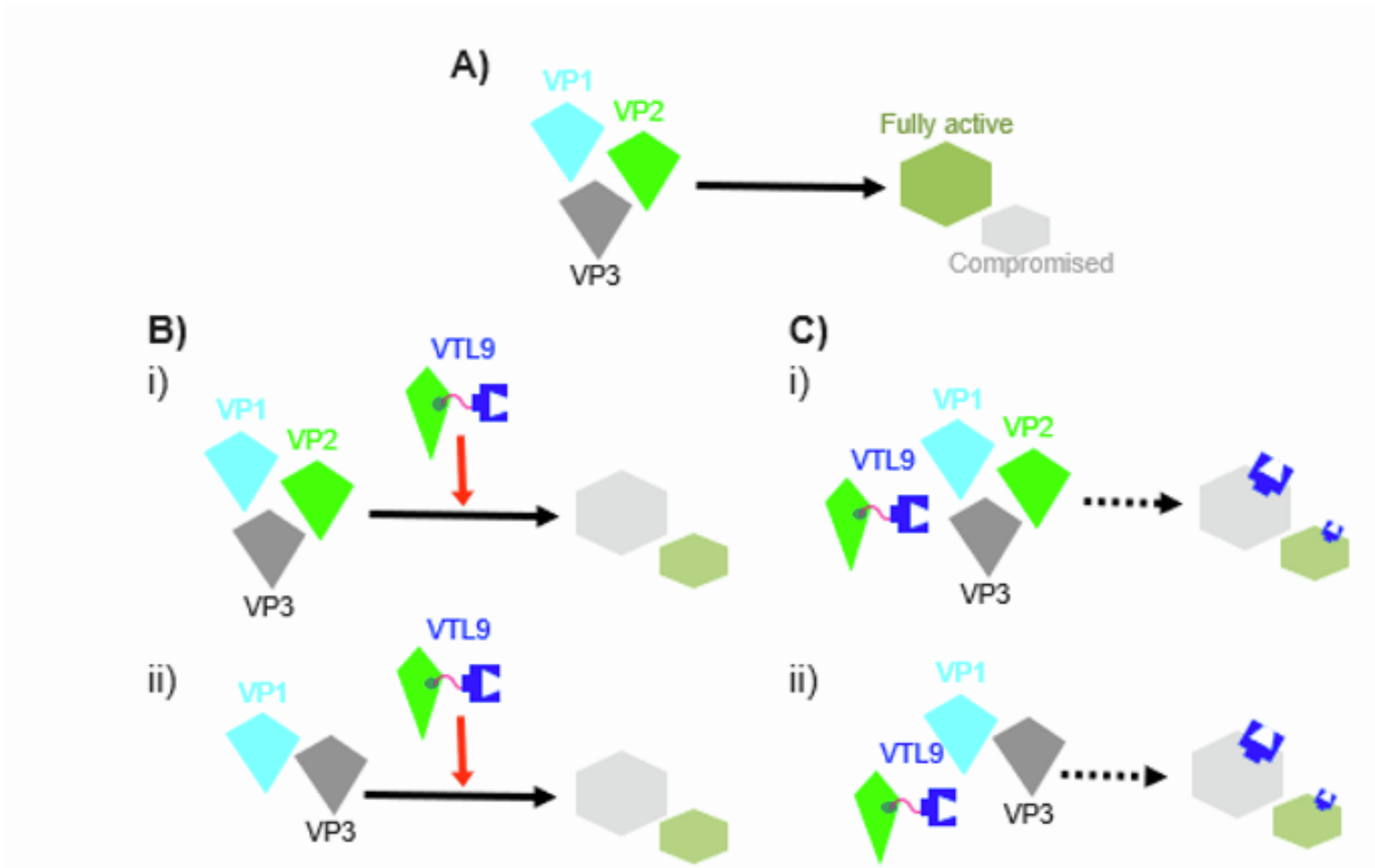

Figure S3. Scenarios whereby VP2-TALE fusion co-expression may impact AAV2 capsid assembly. A) Cartoon representation of VP1, VP2 and VP3 protein association leading ultimately to formation of intact, infection-competent capsids, as green, filled hexagons labelled, 'Fully active', and capsids with compromised formation such that they are non-infectious, as grey filled hexagons labelled 'Compromised'. **B) i)** Representation of the presence of VTL9 inhibiting (red, flat-ended arrow) formation of VP1, VP2 and VP3 into fully active capsids, but without itself being incorporated into capsids. **ii)** As in panel B) i) but without VP2. **C) i)** As in panel B) i) but VTL9 associates with VP1, VP2 and VP3 to form part of resulting capsids, but in a manner that reduces level of fully active capsids (dotted black arrow). **ii)** As in panel C) i) but without VP2.

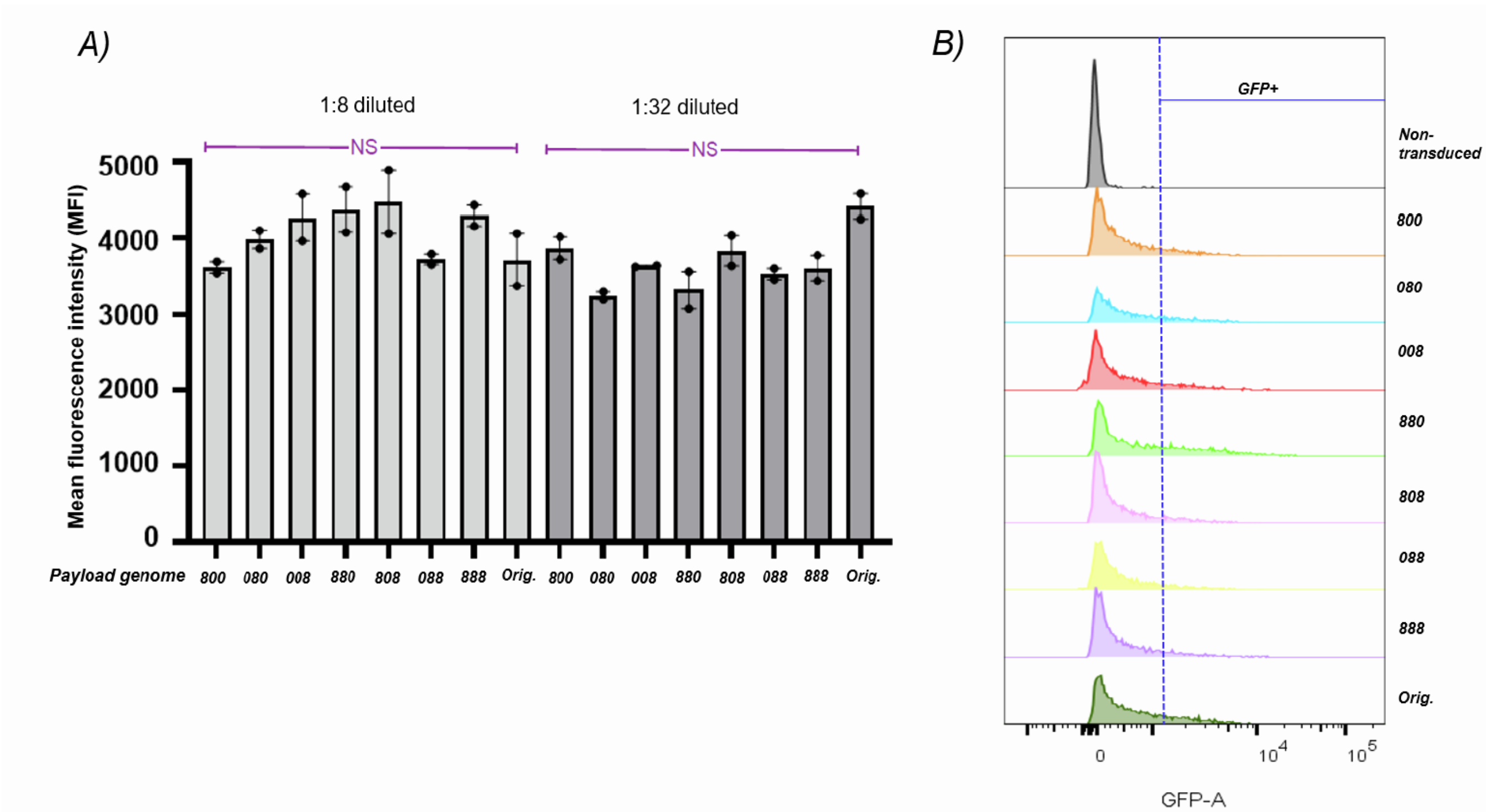

Figure S4. Mean fluorescence intensity (MFI) in target cells as a function of number and location of TALE8 target sequence(s) within payload plasmid. AAV2 samples were produced with engineered payload plasmids 800, 080, 008, 880, 808, 088 and 888, or original, unmodified payload plasmid, pscAAVGFP (data bars labelled 'Orig.'). A) MFI measured from GFP-positive single HEK293T cells after transducing with 1:8 and 1:32 diluted AAV samples. Resulting MFI for each transfection represents mean ± SEM from two biological replicates. Brown-Forsythe ANOVA and Dunnett's T3 post hoc tests were applied to determine statistically significance between all pairs of values. All resulting *p* values were > 0.05 and therefore deemed to be non-significant (NS), so all data was placed under a bracket labelled 'NS'. B) Representative histograms of single HEK 293T cells profiles after transducing with 1:8 diluted AAV samples. The y-axis shows cell count, and the x-axis shows fluorescence intensity. The dotted line shows the gating strategy applied to non-transduced single cell samples to isolate GFP-negative populations and GFP-positive populations.

Table S1. Mass and molar ratio values of plasmids used in transfections in this study. Mass information underlying molar ratios used in transfections that feature in the three plasmid and four-plasmid transfections used in this study. Across all five four-plasmid transfection conditions (A–E), the masses of both Helper and Payload plasmids varied by less than 7% of their respective mean values.

Plasmid Type	Helper	Payload	RepCap or RCdel	Fusion variant	Total mass
**Transfection type**	**Three-Plasmid**				
Mass (ng)	853.7	240.7	405.6	0	1500
Molar ratio	1	1	1	0	
**Transfection type**	**Four-Plasmid A**				
Mass (ng)	701.1	197.6	333.1	268.1	1500
Molar ratio	1	1	1	1	
Transfection type	**Four-Plasmid B**				
Mass (ng)	685.4	195.0	488.5	131.1	1500
Molar ratio	1	1	1.5	0.5	
**Transfection type**	**Four-Plasmid C**				
Mass (ng)	715.7	203.6	170.0	410.6	1500
Molar ratio	1	1	0.5	1.5	
**Transfection type**	**Four-Plasmid D**				
Mass (ng)	676.8	192.6	578.9	51.8	1500
Molar ratio	1	1	1.8	0.2	
**Transfection type**	**Four-Plasmid E**				
Mass (ng)	725.4	206.4	68.9	499.3	1500
Molar ratio	1	1	0.2	1.8	
